# The role of autophagy in cancer: from molecular mechanism to therapeutic window

**DOI:** 10.3389/fimmu.2025.1528230

**Published:** 2025-04-03

**Authors:** Pooya Jalali, Arvin Shahmoradi, Amir Samii, Radman Mazloomnejad, Mohammad Reza Hatamnejad, Anwaar Saeed, Afshin Namdar, Zahra Salehi

**Affiliations:** ^1^ Basic and Molecular Epidemiology of Gastrointestinal Disorders Research Centre, Research Institute for Gastroenterology and Liver Diseases, Shahid Beheshti University of Medical Sciences, Tehran, Iran; ^2^ Department of Laboratory Medicine, Faculty of Paramedical, Kurdistan University of Medical Sciences, Sanandaj, Iran; ^3^ Department of Hematology and Blood Transfusion, School of Allied Medical Sciences, Iran University of Medical Sciences, Tehran, Iran; ^4^ Division of Molecular Medicine, Department of Anesthesiology and Perioperative Medicine, David Geffen School of Medicine, University of California, Los Angeles, Los Angeles, CA, United States; ^5^ Department of Medicine, Division of Hematology and Oncology, University of Pittsburgh Medical Center, Pittsburgh, PA, United States; ^6^ Program in Cell Biology, The Hospital for Sick Children Peter Gilgan Centre for Research and Learning, Toronto, ON, United States; ^7^ Department of Hematology, Oncology and Stem Cell Transplantation Research Center, Research Institute for Oncology, Hematology and Cell Therapy, Tehran University of Medical Sciences, Tehran, Iran

**Keywords:** autophagy, cancer, immunotherapy, precision-medicine, tumorigenesis

## Abstract

Autophagy is a cellular degradation process that plays a crucial role in maintaining metabolic homeostasis under conditions of stress or nutrient deprivation. This process involves sequestering, breaking down, and recycling intracellular components such as proteins, organelles, and cytoplasmic materials. Autophagy also serves as a mechanism for eliminating pathogens and engulfing apoptotic cells. In the absence of stress, baseline autophagy activity is essential for degrading damaged cellular components and recycling nutrients to maintain cellular vitality. The relationship between autophagy and cancer is well-established; however, the biphasic nature of autophagy, acting as either a tumor growth inhibitor or promoter, has raised concerns regarding the regulation of tumorigenesis without inadvertently activating harmful aspects of autophagy. Consequently, elucidating the mechanisms by which autophagy contributes to cancer pathogenesis and the factors determining its pro- or anti-tumor effects is vital for devising effective therapeutic strategies. Furthermore, precision medicine approaches that tailor interventions to individual patients may enhance the efficacy of autophagy-related cancer treatments. To this end, interventions aimed at modulating the fate of tumor cells by controlling or inducing autophagy substrates necessitate meticulous monitoring of these mediators’ functions within the tumor microenvironment to make informed decisions regarding their activation or inactivation. This review provides an updated perspective on the roles of autophagy in cancer, and discusses the potential challenges associated with autophagy-related cancer treatment. The article also highlights currently available strategies and identifies questions that require further investigation in the future.

## 1 Introduction

Cancer constitutes a critical global health and economic challenge, with projections indicating an intensification in the coming years. The prevalence of cancer surpassed 18 million cases in 2018 and is estimated to escalate to nearly 29 million cases by 2040, primarily due to an aging and growing population. As of 2022, approximately 2 million new cancer cases had been diagnosed, amounting to around 5,500 new cases per day (1). Despite considerable efforts towards prevention and treatment, cancer continues to be the leading cause of death and poses a substantial burden on healthcare systems worldwide.

Despite significant advancements in the field of cancer therapy, there are still numerous challenges that hinder successful treatment outcomes. These challenges primarily arise from an incomplete understanding of the precise mechanisms underlying cancer pathogenesis. Therefore, it is imperative to gain a comprehensive understanding of the physiological and pathophysiological processes occurring at the cellular and molecular levels in order to effectively manage the disease and address its associated complications. Research exploring the mechanisms of cancer initiation has revealed that the accumulation of genetic, epigenetic, and metabolic alterations contributes to the development of malignant cells, ultimately leading to cancer cell invasion and the emergence of drug resistance (2, 3).

In recent years, targeted therapies, particularly cancer immunotherapy, have emerged as promising clinical approaches that have significantly improved overall survival rates for cancer patients (4, 5). Consequently, there has been a growing focus on investigating the role of the immune system in developing effective strategies for cancer diagnosis and immunotherapy-based treatments (6, 7). Given the inherent heterogeneity of cancer, along with variations in the tumor’s site of onset and the composition of immune cells, the precise selection of immunotherapies to specifically target tumor cells becomes imperative.

Autophagy has recently garnered attention as a cellular process that plays a pivotal role in modulating tumorigenesis, acting either as a tumor suppressor or promoter. Autophagy is a catabolic cellular degradation response that is triggered by starvation or stressful conditions. It involves the encapsulation, digestion, and recycling of cellular proteins, organelles, and cytoplasm to sustain cellular metabolism (8, 9). There are three main types of autophagy: macroautophagy, microautophagy, and chaperone-mediated autophagy (CMA). Basal autophagy is crucial for preserving cellular homeostasis, serving as a quality control mechanism for proteins and organelles. This process operates in parallel with the ubiquitin-proteasome degradation pathway, preventing the accumulation of polyubiquitinated and aggregated proteins (10–15). Additionally, autophagy plays a role in pathogen removal (16) and apoptotic cell digestion (17). One of autophagy’s critical mechanisms is the execution of an intracellular degradation pathway mediated by double-membrane vesicles called autophagosomes. While general autophagy aggregates cytoplasmic components within autophagosomes and delivers them to lysosomes for degradation, selective autophagy specifically targets damaged organelles, protein aggregates, and intracellular pathogens (18). Disruptions or mutations in ATGs, which regulate the autophagic process, have been implicated in various human diseases, including neurological disorders, autoimmune diseases, metabolic disorders, infectious diseases, and cancer (19–21). Therefore, identifying autophagy mediators and related metabolic pathways is critical for understanding autophagy’s role in tumorigenesis (22).

Autophagy has been a subject of cancer research for several years, with numerous studies demonstrating its association with cancer onset and treatment (23, 24). In particular, autophagy has been shown to regulate various oncogenes and tumor suppressor genes, as evidenced by multiple studies (25, 26). Despite extensive research efforts, the role of autophagy in cancer remains enigmatic and controversial. While some studies suggest that autophagy promotes tumorigenesis, others argue that it inhibits cancer development (27–30). Furthermore, autophagy has displayed dual roles as a pro-metastatic or anti-metastatic effector (31). In the early stages of cancer metastasis, autophagy inhibits metastasis by limiting cancer necrosis, inflammation responses, and reducing cancer cell invasion and migration. However, in advanced stages of metastasis, autophagy plays a pro-metastatic role by promoting cancer cell survival (32, 33). Consequently, the function of autophagy in cancer remains a complex and ongoing area of investigation. In the cancer microenvironment, autophagy provides cellular energy and inhibits cytotoxicity under stressful conditions (34, 35).

Since autophagy impacts tumor cells at various stages (initiation, development, and progression) with conflicting roles, it remains a complex phenomenon concerning cancer treatment. Current evidence suggests that tumor cells may use autophagy as a protective shield to resist numerous anticancer therapies (36). In line with these findings, autophagy suppression has been shown to enhance the benefits of cancer therapies by sensitizing tumor cells to these drugs (37–39). Additionally, autophagy may play a crucial role in maintaining stemness in cancer stem cells and regulating their homeostasis (40, 41). Thus, elucidating the molecular mechanisms of autophagy can help manipulate this process for the benefit of cancer therapy and clarify which strategies should be adopted for clinical interventions to achieve desirable outcomes.

**Figure 1:**
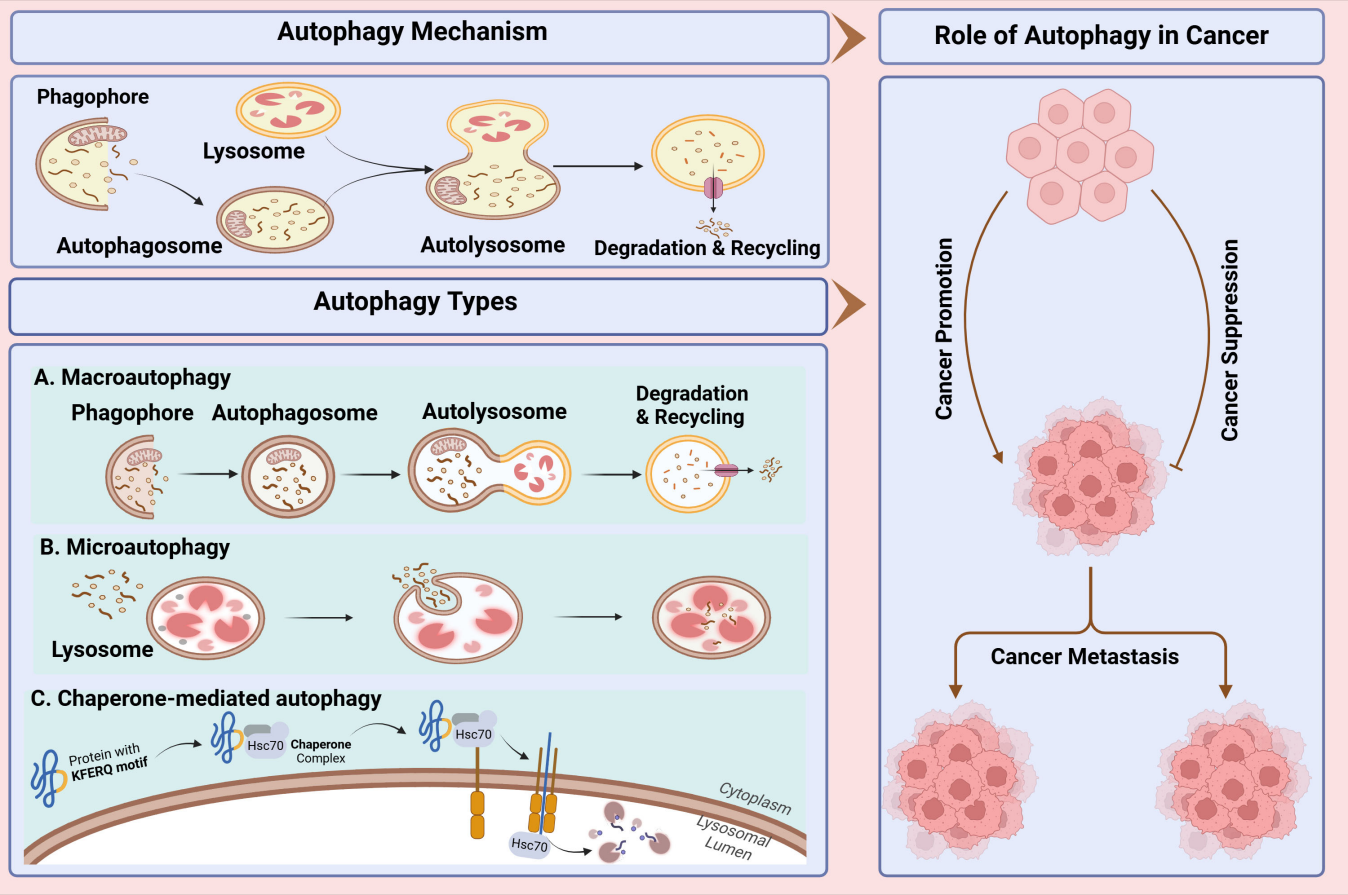
Study workflow for comprehensive study of autophagy and its role in cancer. In this study, first, we present autophagy mechanism and types of autophagy then its dual role in cancer including progression, metastasis and suppression of cancer cells discussed. Lastly, we discussed the current landscape of autophagy inhibitors and the therapeutic approaches employed to regulate autophagy.

In this review, we aim to present current knowledge on the role of autophagy in cancer and its effects on suppressing or promoting malignancies through interactions with immune system components. By elucidating the role of autophagy in tumor suppression and growth, we seek to provide valuable insights into the context-dependent implementation of therapeutic strategies. Subsequently, we will explore the potential of anticancer therapies based on modulating autophagy as either an inhibitor or promoter. Lastly, we will discuss the current landscape of autophagy inhibitors and the therapeutic approaches employed to regulate autophagy (**Figure 1**).

## 2 The autophagy types

Autophagy is a cellular process through which both intracellular and extracellular substrates are delivered to lysosomes for degradation (42). This process is required to maintain cellular homeostasis (43), produce amino acids for sustained viability during periods of starvation (44, 45), and increase protection against pathogens (46). Based on the delivery route and cargo specificity, three different types of autophagy are distinguished: macroautophagy, microautophagy, and chaperone-mediated autophagy (CMA) (43). Both macro- and micro-autophagy include the dynamic rearrangement of membranes to cover parts of the cytoplasm and have the capacity to sequestrate large structures such as whole organelles.

**Figure 2:**
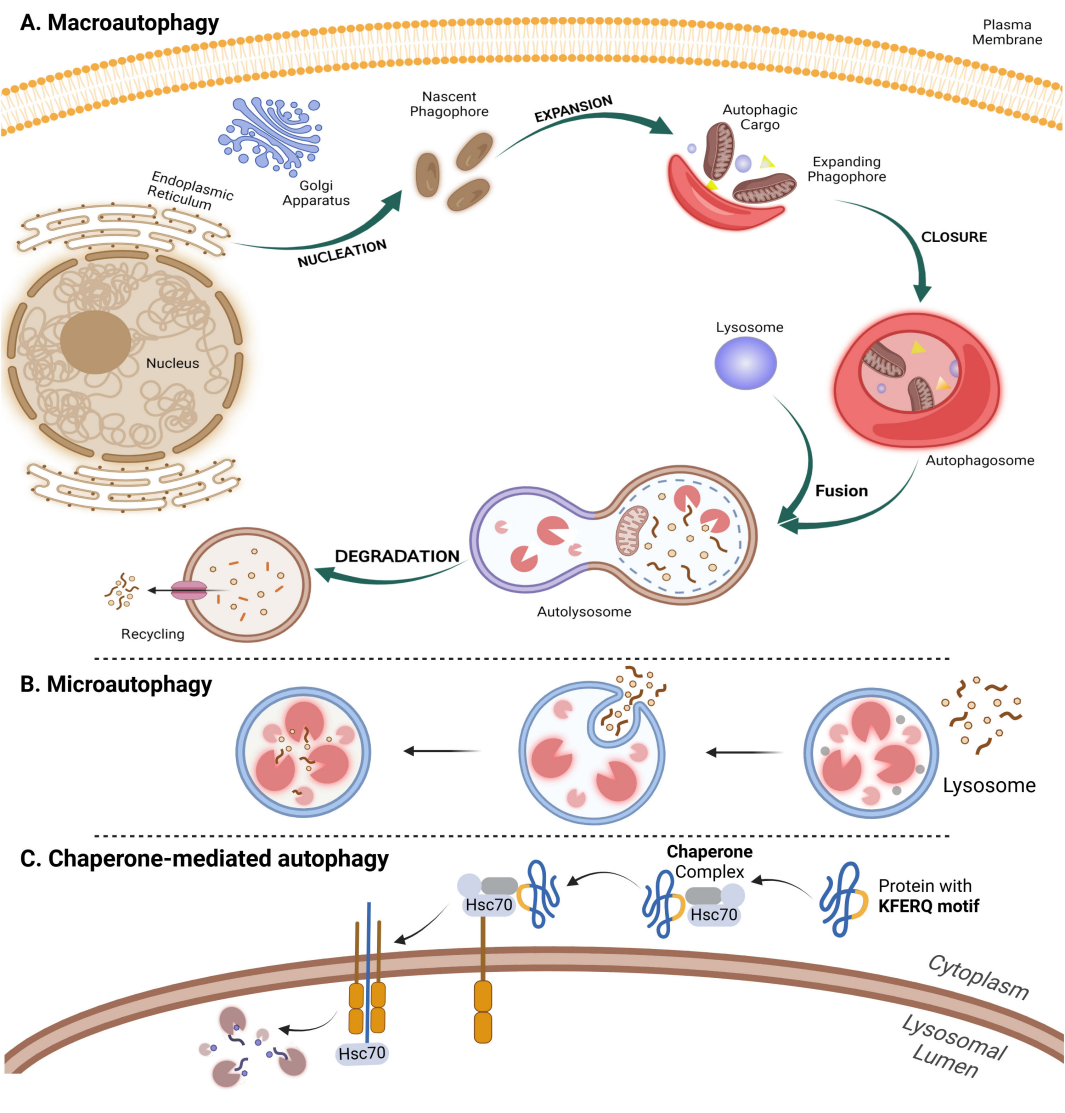
The autophagy mechanism and its three types. The process of autophagy is divided into five distinct stages: initiation, nucleation, expansion and elongation, closure and fusion, and cargo degradation. Once autophagy is induced, ATG proteins are gathered to form autophagy initiation complex. Subsequently, recruitment of other ATG proteins leads to the generation of phagophore which is then matured as a bilayer membrane structure called autophagosome. When autophagosome fuses with lysosome to form autophagolyosome, cellular components undergo enzymatic degradation to be reused by cells. **(A)** Macroautophagy involves the formation of autophagosome as a double-membrane vesicle that engulfs cytoplasmic material. The autophagosome then fuses with a lysosome in order to degrade and recycle the sequesteredcontents. **(B)** In microautophagy, lysosomes directly engulf and digest cytoplasmic materials, such as damaged organelles and misfolded proteins. **(C)** Chaperone-mediated autophagy (CMA) occurs when specific proteins, containing a KFERQ motif, are selectively recognized by chaperones and transported to the lysosome for degradation.

Macroautophagy involves the sequestration of cytoplasmic components within a *de novo* double-membrane vesicle called the autophagosome. The autophagosome subsequently fuses with the lysosome or vacuole, releasing its inner single-membrane vesicle into the lumen. The autophagic body membrane is then lysed, allowing the contents to be broken down and the resulting macromolecules to be transported back into the cytosol through membrane permeases for reuse (47) (**Figure 2A**). Microautophagy is a process by which the cytoplasm is directly engulfed by the lysosome through invagination, protrusion, and septation of the lysosomal membrane (48) (**Figure 2B**). Unlike microautophagy and macroautophagy, which can non-specifically engulf bulk cytoplasm, CMA selectively targets specific proteins linked to a KFERQ pentapeptide motif. This targeting motif is commonly found in all CMA substrates, making CMA highly specific in its selection of proteins for degradation (49) (**Figure 2C**).

Autophagy was originally defined as a pathway for bulk degradation that is triggered by glucagon and nutrient scarcity (50, 51). This type of bulk autophagy functions to recycle essential building blocks to make up for a deficiency in nutrients and is generally considered non-selective regarding its substrates, referred to as cargos (52, 53). However, it has become evident that autophagy also plays a vital role in maintaining intracellular homeostasis in cells that are not nutrient-deprived, as it selectively degrades various cargo materials including aggregated proteins, damaged mitochondria, excess peroxisomes, and invading pathogens (54–56). The significance of selective autophagy for cellular homeostasis is underscored by research showing that tissue-specific deletion of autophagy-related genes in mice leads to conditions such as neurodegeneration or liver cancer (10, 57–59). Furthermore, studies have indicated that cells with impaired autophagy are unable to eliminate certain intracellular pathogens (60, 61). Recent evidence also suggests that selective autophagy is crucial for regulating intracellular free iron levels by modulating the amounts of the iron-binding protein ferritin, a process known as ferritinophagy (62–65). Also, the selective autophagy of the endoplasmic reticulum is referred to as “ER-phagy/reticulophagy” (66), and the degradation of ribosomes is called “ribophagy” (67).

In general, autophagy is an essential, ubiquitous, evolutionary, catabolic, and self-destructive process that mediates the elimination of cytoplasmic macromolecules to maintain genomic integrity, achieve cellular metabolism, and ensure cell survival (46, 68–70). It is a natural regulatory mechanism that retains beneficial substances and removes harmful substances from the body while playing a housekeeping role in eliminating misfolded or aggregated proteins, destroying damaged organelles, proteins (71–73), and cancerous substances (18), and eliminating foreign pathogens such as viruses through a destructive lysosomal pathway (58, 74–76).

Studies have shown that autophagy dysfunction is associated with the accumulation of damaged proteins and organelles, which can contribute to a variety of diseases, including cancer, neurodegeneration, and metabolic disorders (58, 77). Additionally, the modulation of autophagy has emerged as a potential therapeutic strategy for such diseases. The potential clinical applications of autophagy modulation are diverse, ranging from cancer therapy to neurodegenerative disease treatment. For example, inducing autophagy has been shown to sensitize cancer cells like colon and breast cancer to chemotherapy (78, 79), while inhibiting autophagy can protect neurons from the toxic effects of misfolded proteins in Alzheimer’s Disease, Parkinson’s disease and Huntington disease (80–83).

## 3 Autophagy-related signaling pathways

Autophagy is a well-regulated process coordinated by several protein complexes acting stepwise. The three most important complexes playing roles in the initiation and elongation of autophagy are (1): the ULK1/2 complex, the principal regulator of autophagy induction (2), class III phosphatidylinositol 3 kinase complex (PI3KC3), which contributes to phagophore nucleation, and (3) Atg5-Atg12/Atg16 complex, which contributes to autophagosome elongation.

**Figure 3:**
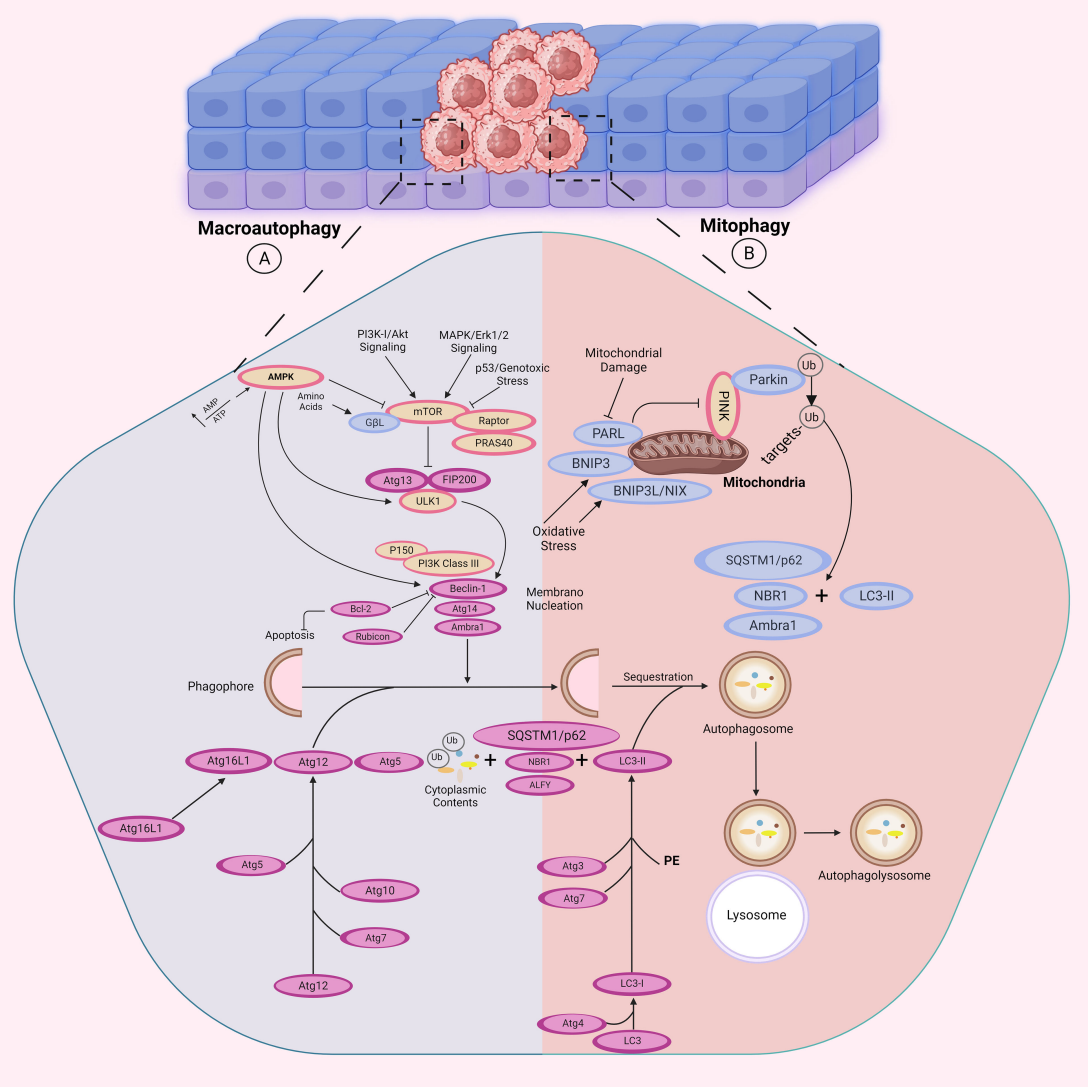
Autophagy-related signaling pathways. The autophagosome formation is mediated by the activities of three complexes: the ULK1, the phosphoinositide 3-kinase catalytic subunit type III (PI3KC3), and the Atg16L1 complexes. **(A)** In response to cellular ATP reduction, AMPK is activated to inhibit mTOR activity. mTOR downregulation leads to autophagy activation by releasing its inhibitory effect on ULK1 complex (ULK1, ATG13, and FIP200). However, in PI3K/Akt and MAPK/Erk signaling pathways, autophagy is suppressed in response to mTOR activity. The initial phagophore formation requires ULK1 complex to activate PI3KC3 complex which is consisted of Atg14 and Beclin-1/Atg6. Binding of Bcl-2 to Beclin-1 inhibits autophagy by preventing the formation of the PI3KC3. PI3KC3 then recruits two ubiquitin-like conjugation systems: The Atg12 (Atg5, Atg7, Atg10, Atg12 and Atg16) and Atg8 (Atg3, Atg4, Atg7 and Atg8) conjugation systems which participate in autophagosome formation, which further fuses with the lysosome for substrate degradation. AMBRA1 is another protein that directly binds to Beclin-1 to promote autophagy induction. **(B)** In response to mitochondrial damage, hypoxia or increased ROS production, mitophagy is triggered as a selective process for degradation of mitochondria by autophagy. NBR1 and BNIP3, BNIP3L/NIX and SQSTM1/p62 function as receptors on the mitochondria outer-membrane. BNIP3 competes with Beclin-1 for binding Bcl-2 and thus releases Beclin-1 for participating in mitophagy. Moreover, p62/SQSTM1 protein mediates clearance of ubiquitinated or aggregated proteins by binding them to the autophagy mediators for degradation. LC3 is involved in phagophore elongation and autophagosome-lysosome fusion.

Of these, the ULK1/2 complex is tasked with sensing upstream signals for autophagy initiation. The complex consists of ULK1, its homolog ULK2, ATG13, ATG101, and FIP200 (RB1CC1). The activity of ULK1 is also tightly controlled by two major nutrient-sensing kinases: AMPK and mTORC1. AMPK activates autophagy by direct phosphorylation and activation of ULK1, whereas mTORC1 inhibits autophagy by phosphorylation of ULK1 at specific sites to inhibit its activation. When activated, the ULK1/2 complex initiates the formation of autophagosomes, augmenting the level of autophagy. When the serine-kinase activity of ULK phosphorylates components of the ULK complex or other members of the core autophagy machinery, it triggers starvation-induced autophagy (84–86). This phosphorylation of components leads to modulation of their catalytic activities and subcellular distribution, which ultimately initiates autophagy. The class III phosphatidylinositol 3 kinase complex (PI3KC3), which contains the Atg14 subunit and Beclin-1/Atg, is another factor that contributes to the initiation and assembly of the phagophore at the onset of autophagy (**Figure 3A**) (87).

In addition, there are two ubiquitin-like conjugation systems that are crucial for the elongation of phagophore. The first system involves the Atg5-Atg12/Atg16 complex, where Atg12 is conjugated to Atg5 and catalyzed by Atg7 and Atg10 (88, 89). The Atg5-Atg12 conjugate then associates with Atg16 via Atg5 and this complex is recruited to the phagophore by WIPI2 (90, 91). The second system is comprised of the Atg8 proteins, which are divided into two sub-families, LC3 and GABARAP (92). LC3-I, which is a cytosolic ubiquitin-like protein, is cleaved by the cysteine protease Atg4 to expose its C-terminal glycine residue. Atg7 and Atg3 enzymes, along with the Atg5-Atg12/Atg16 complex, then conjugate LC3-I to phosphatidylethanolamine (PE) to form LC3-II, which leads to the formation of autophagosome. Subsequently, autophagosome is fused with lysosome to degrade the sequestered substrates (89, 93, 94).

Selective autophagy or cargo-induced autophagy is a non-starvation, degradative process that has a role to play in cellular homeostasis through the specific degradation of individual cellular components (56). Excluding mitophagy, all other types of selective autophagy in the other categories of selective autophagy also play important roles to ensure cell homeostasis. Ferritinophagy aids in intracellular iron homeostasis by degrading ferritin inside lysosomes. The cargo receptor NCOA4 binds ferritin and facilitates its interaction with autophagosomes, facilitating the release of iron and maintaining cellular balance (62–66). ER-phagy specifically degrades damaged or redundant endoplasmic reticulum (ER) components to maintain ER homeostasis. Receptors such as FAM134B and RTN3 interact with LC3 to facilitate autophagic sequestration and degradation of ER fragments under stress conditions (62–66). To recognize specific cargoes, selective autophagy employs cargo-receptor proteins that are identifiable to the machinery of autophagy. BNIP3L (Nix) is one such protein referred to as Nix that functions as a receptor in mitochondria with recent reports identifying its function to mediate mitophagy. Through the interaction with LC3 via the N-terminal interacting region, Nix plays a role in regulating this process (95).

Mitophagy, a form of selective autophagy, degrades faulty mitochondria to help maintain cellular homeostasis. PINK1 is enriched on faulty mitochondria and recruits Parkin, which facilitates their ubiquitination and subsequent degradation by autophagosomes (96).

Moreover, several recent studies have revealed that the ubiquitin-binding protein p62/SQSTM1 serves as a receptor that links the autophagy machinery to different cargo targets, such as ubiquitinated protein aggregates and bacterial pathogens (97). This protein interacts with LC3 and ubiquitinated cargo through its LC3-interacting region and C-terminal ubiquitin-associated (UBA) domain, respectively (97, 98). Another ubiquitin-binding protein, called NBR1 (neighbor of BRCA1 gene 1), shares similar domain organizations with p62/SQSTM1, and it directly interacts with p62/SQSTM1. NBR1 also functions as a receptor for selective autophagy targeting of ubiquitinated protein aggregates (99) (**Figure 3B**).

## 4 Autophagy: from molecules to cancer

The better understanding of autophagy indicates the ethological relevance of ATG mutations to various cancers. Ultimately, modeling revealed that while autophagy is an anti-tumorigenic process, excessive autophagic activity would have also been linked to neurodegenerative diseases -consistent with the duality that autophagy embodies in health and disease (100).

Autophagy was initially considered as a tumor-suppressor mechanism. Links that connect autophagy to tumors stem from two meager pieces of evidence (101). First, it was found that BECN1, the gene encoding Beclin-1 and the yeast ortholog Atg6, is monoallelically eliminated in breast, ovarian, and prostate cancers (102, 103). It has also been demonstrated that BECN1 deficient mice are more susceptible to develop hepatocellular and lung carcinomas as well as lymphomas (57, 104). However, induction of deficient autophagy through deletion of Atg5 or Atg7 in mice leads to benign liver tumors, indicating that autophagy could play an essential role in inhibiting tumor initiation in liver, which necessitates further research in human cancers (105). In addition, ectopic overexpression of BECN1 in human breast cancer cell line (MCF7 cells), which have very low levels of endogenous Beclin-1, resulted in activation of autophagy, which concurred with reducing proliferation and inhibiting tumorigenesis (103).

Beclin-1 is a highly investigated mammalian-specific autophagy regulator that shares homology with yeast Atg6. It serves as a crucial platform for the recruitment and initiation of PI3KC3 (106). Under normal conditions of mammalian cell growth, Beclin-1 associates with Bcl-2, an anti-apoptotic protein, through an interaction with the Beclin-1 BH3 domain. This interaction inhibits the formation of the Beclin-1/PI3KC3 complex and suppresses autophagy (36). Conversely, exposure to nutrient-deficient conditions disrupts the Beclin-1/Bcl-2 complex, releasing Bcl-2 from Beclin-1 and subsequent autophagy induction (107).

Consistently, ectopic overexpression of BECN1 in colon cancer cell lines leads to growth inhibition, which is attributed to the low endogenous expression of this gene in these cancer cells (108). Other mutations in ATGs such as Atg2B, Atg5, Atg9B, Atg12, and UVRAG have been proved to be associated with gastric and colorectal cancers beyond the BECN1 (109), therefore offering further support to the roles of autophagy in tumor suppression.

Adding to the support of autophagy’s role in cancer, experimental studies in genetically modified mouse models have shown that the loss of key regulators of autophagy increases the incidence of tumors. It was found first in mice hemizygous for BECN1 and later in mice lacking Atg4C and BIF1 that lack of these autophagic factors could lead to increased tumor formation incidence (102, 103, 110). Similarly, in another study, Takamura et al. reported that mice with Atg5 and Atg7 deficiency are more susceptible to develop liver tumor, which could be reversed by concomitant knockout of the p62 gene (57). Collectively, these observations confirmed that autophagy is required for tumor suppression.

Atg5 is a crucial autophagosome-forming protein that acts as an E1-activating enzyme in eukaryotic cells (111, 112). E1-activating enzymes are known as ubiquitin-activating enzymes which are responsible for catalyzing proteins undergoing ubiquitination reaction (111, 112). Atg5 combines with Atg12 through an ubiquitin-like system. Alongside LC3-II, which is a key molecule in autophagosomal membrane formation via the Atg5-Atg12/Atg16 complex, Atg5 also plays an essential role in various biological processes such as viral infection (113, 114), tumor apoptosis (115, 116), and tumor proliferation (117, 118). Several autophagy-specific regulatory genes have been classified in yeast, including several genes with mammalian homologs. Among them, Atg5, a key regulator of autophagosome formation, is one of the most studied ATGs. Furthermore, Atg5 also participates in the regulation of cell death. Ectopic expression of Atg5 can stimulate cells to undergo apoptosis in response to apoptotic stimuli, such as anticancer agents (116). In addition, during cell death, calpain can break down Atg5, and truncated Atg5 leads to mitochondrial-dependent apoptosis (119). This suggests that Atg5 may act as a critical regulator in determining whether cells undergo autophagy or apoptosis.

Another vital component of stress signaling and adaptation is the tumor suppressor p53. A wide range of stressors, such as DNA damage, metabolic stress, and oxidative stress, activate P53 (120). In response to these stressors, p53 regulates gene transcription or acts through non-transcriptional mechanisms to cope with stress adaptation (e.g., cell cycle arrest) or to destroy cells that cannot be repaired by apoptosis or aging. One component of this p53-mediated transcriptional response is autophagy activation (120). Conversely, autophagy has the ability to suppress p53 levels and functions. The p53 and autophagy pathways are intricately intertwined and exert significant influence on stress, metabolism, and cancer responses (120).

The regulation of autophagy can influence the expression of tumor suppressor proteins or oncogenes. Furthermore, autophagy modulation by certain anticancer medications can contribute to either the survival or destruction of cancer cells (29, 30). Mammalian target of rapamycin (mTOR) and AMP-activated protein kinase (AMPK) negatively regulate tumor suppressor factors, which induces autophagy and suppresses cancer onset (121). Conversely, mTOR Class I PI3K and AKT activate oncogenes, suppressing autophagy and promoting cancer formation (122).

Numerous preclinical investigations have demonstrated that targeted treatments and DNA damaging agents have the potential to induce autophagy. However, the majority of studies have found that the autophagy elicited by these anticancer drugs is cytoprotective rather than cytotoxic (123). Currently, some specific autophagy inducers have been introduced. Most agents that induce autophagy either hinder other crucial cellular functions, such as mTOR signaling, or activate additional stress responses, such as the unfolded protein response (124). Tat-Beclin1 is a fusion peptide believed to act as an inducer of autophagy, though the precise mechanism remains undefined (125).

Compound 5e is a newly synthesized fluorescent molecule and a selective mTOR inhibitor by interacting with FKBP12 reducing tumor growth in human non-small cell lung cancer cells. This inhibition subsequently induces autophagy, suggesting that autophagy is a secondary response and not the primary mechanism of action (126). Additionally, some other natural herbal derivatives with anti-tumor activities have been introduced to induce autophagy in cancer cells. Honokiol and Isobavachalcone, respectively, both antitumoral in effect, were later shown to have autophagy as a secondary response in melanoma and myeloma cells. Honokiol targets the Notch signaling pathway, reducing the stemness of melanoma. In contrast, apoptosis triggered by the isobavachalcone was achieved through the activation of caspases and increased LC3-II expression, linking autophagy with death mechanisms of cells (127, 128). HNK is a natural compound that targets notch signaling pathway to maintain melanoma cells stemness and self-renewal via inhibiting the expression of downstream target proteins including Hes-1 and cyclin D1. Furthermore, the treatment of H929 myeloma cells with IBC enhances the expression of LC3-II which is an autophagosome formation marker. IBC-induced cell death is triggered by the activation of apoptosis mediators such as caspases 3,9 and the cleavage of poly ADP-ribose polymerase (PARP) and the proteolytic activation of protein kinase C (PKC) (127, 128). These inducers may be effective for reducing the development of benign lesions such as polyps, but further research is required to find the appropriate targets and chemical agents that may specifically induce autophagy.

There is increasing evidence that inhibiting autophagy may be an effective treatment for advanced cancers (129). Additionally, several research groups have demonstrated that autophagy might support tumor immunity by serving as a tumor protector. For example, RAS proteins are small GTPases that regulate key signaling pathways for metabolism, cell survival, and proliferation. In cancer cells with RAS mutations, autophagy is often upregulated as a means of supporting tumor survival and progression (18, 130–132). The formation of some lethal malignancies, such as lung, colon, and pancreatic, is linked to the occurrence of RAS-activating mutations, which induce autophagy, which promotes tumor development, survival, and oncogenesis (18, 133–136).

Genetically engineered mouse models (GEMMs) studies have shown that autophagy suppresses the formation of early-stage benign tumors but facilitates the progression of advanced malignancies in mouse models of lung adenocarcinoma and pancreatic ductal adenocarcinoma (PDAC) driven by mutant RAS or BRAF (137–141). Similarly, it was shown in a mouse model of breast cancer that suppression of autophagy by FIP200 inhibits tumor initiation and progression (142). The activation of necrotic cell death and an inflammatory response in tumors with autophagy and apoptosis deficiencies partially explains how the loss of autophagy’s pro-survival role promotes carcinogenesis (143). Preventing starvation-induced survival through autophagy and rerouting apoptosis-defective tumor cells toward a necrotic cell fate can result in the development of chronically necrotic tumors. This process may impair the normal wound-healing response and promote tumor development, suggesting that autophagy defects represent a non-cell-autonomous mechanism for promoting tumorigenesis (144–146).

In contrast to apoptosis, necrosis and cell lysis lead to the release of nuclear high mobility group box 1 (HMGB1) from cells, which stimulates the innate immune response, the recruitment of inflammatory cells, cytokine production, and nuclear factor-B (NF-B) activation. In certain cases, these events are associated with increased tumorigenesis (147–149). Inhibition of autophagy by constitutively activating AKT in apoptosis-defective cells results in necrosis in response to metabolic stress *in vitro*, and *in vivo*, necrosis coincides with NF-B activation and promotes tumorigenesis (143).

Despite significant investment into this field, the detailed molecular mechanisms by which autophagy drives cancer progression still remain elusive and are therefore poised for further work. Therefore, further studies are needed to investigate the relationship between different processes of cell death and their impact on the immune system and the tumor microenvironment to better understand their relationship and regulate tumor growth. In the following, the dual role of autophagy in tumor promotion and suppression will be discussed.

## 5 Cancer immunity in autophagy

The primary role of the immune system is to defend the host against external threats like bacteria and toxins and maintain the body’s structural integrity (150). The categorization of the immune response into two distinct parts, namely innate immunity, which provides non-specific resistance to infections, and adaptive immunity, which targets particular pathogens through a highly specialized and adaptable process, has been artificially established (150). Both innate and adaptive immune responses require autophagy, a cellular mechanism, to function properly (29). The progressive and in-depth analysis of the molecular underpinnings of cancer progression, carcinogenesis, and the dissemination of cancer cells to other parts of the body has resulted in more precise, efficacious, and specialized treatment strategies for various forms of solid and hematological malignancies, particularly those with a high propensity for distant metastasis (151). Meanwhile, the recognition of various pathways implicated in the advancement of cancer or, conversely, in the elimination of tumors, emphasized the crucial significance of the immune reaction (151). Researchers can develop more effective cancer treatments by having a better understanding of the autophagy concept, its dual functions, and how it interacts with the immune system.

The complement system and innate immunity are invariably triggered by inflammation, the body’s first line of defense (152). On the other hand, when pathogens or pathogenic peptides are captured and presented by antigen-presenting cells (APCs), such as macrophages, B-cells, or antigen-presenting dendritic cells (DC) they activate T-lymphocytes, which in turn induce cell death (153). Moreover, the promotion of the infiltration of DCs and the recruitment of CD4+ helper and CD8+ cytotoxic T lymphocytes to the tumor microenvironment is seen (154). Additionally, autophagy in dying tumor cells is essential for the induction of immunogenic cell death, which enables the effective detection of tumors by the immune system (6, 155). Major histocompatibility complex (MHC) class II molecules present antigenic, foreign proteins on antigen-presenting cells, activating CD4+ T-cells and CD8+ T cells, and natural killer (NK) cells create cytolytic granules, which autophagy encourages destruction (29). The cells of many bodily organs occasionally rely on autophagy to enhance their performance. For example, thymic epithelial cells need autophagy to recognize host and foreign antigens via MHC class II (29).

Neoplastic cells exhibit a panel of T-cell-recognizable antigens (156). Tumor antigen presentation is a vital part of anticancer reactions, and a malfunction in this system may lead to tumor leakage from immune surveillance, a factor typically linked with cancer expansion, and inhibiting autophagy can impede the process (156, 157).

A number of investigations have initiated the process of defining the distinct regulation mechanisms of macroautophagy and CMA in peripheral T cells. Initial research has indicated that the induction of macroautophagy in T cells involves specific signals. It has been stated that CD4+ and CD8+ T cells exhibit upregulation of macroautophagy upon engagement of the T cell receptor (TCR) (158–161). The signaling mechanisms underlying the stimulation of macroautophagy in active T cells are still being fully understood (158). The activation of the mitogen-activated protein kinase (MAPK) JNK, located downstream of the TCR, has been suggested to play a role in the initiation of macroautophagy (15). This is supported by findings indicating that the inhibition of JNK1 or JNK2 through chemical means or genetic deletion results in a reduction in activation-induced macroautophagy in CD4+ T cells (15). None of these processes, however, have been implicated in the activation of autophagy in T cells as of yet (158). The upregulation of the expression of the lysosomal associate membrane protein 2A (LAMP-2A) in T cells after TCR engagement is in response to the enhanced production of reactive oxygen species in activated cells (162). The production of ROS in CD4+ T cells is regulated by intracellular calcium signaling, which has the transcription factor nuclear factor of activated T cells (NFAT) as one of its key targets (163–165).

Macroautophagy is crucial for preserving the homeostasis of T cells (158). Since T cells must significantly reduce their mitochondrial content as they develop from immature peripheral naive T cells into single-positive thymocytes, mitophagy-regulated mitochondrial recycling is particularly crucial in T cells (158). In T cells lacking essential ATG proteins, organelle turnover—including those of the mitochondria and endoplasmic reticulum—is severely impaired (166–168). Proapoptotic protein levels may have increased in T cells due to increased oxidative stress as well as a potential role for autophagy in the turnover of a number of those proteins and this would also increase the likelihood of cell death, which occurs regardless of functional macroautophagy (160, 169).

According to a number of studies, T cells lacking the crucial Atg genes have lowered proliferative reactions to TCR engagement resulting in being unaffected by CD28 or IL2-receptor signaling (158). Defects in activation-induced proliferation are also seen as autophagy is acutely blocked using inducible deletion of ATG genes or using chemical inhibitors, both of which are sure to have a negative effect on the responses to antigens due to the altered metabolic output and mitochondrial dysfunction seen in T cells from Atg-deficient mice (161). In autophagosomes located in resting cells, organelles, and particularly mitochondria, seem to be the preferred cargo. Conversely, in autophagosomes observed in activated cells, cytosolic material is favored over organelles (161). This implies that selective cargo degradation may be involved in the control of T-cell activation-induced responses (158). DeVorkin et al. demonstrated that the elimination of Atg5 or Atg7 in T cells resulted in a remarkable denial of tumor implants in syngeneic mouse tumor models (170). Research conducted by Mgrditchian and colleagues has demonstrated that the suppression of BECN1 gene expression enhances the infiltration of T cells to the immune system’s microenvironment (171).

In addition to macroautophagy, CMA functions in antigen presentation, especially cytoplasmic antigens (153, 172). Antigen processing may include more than one kind of autophagy (153). Antigens produced from outside the cell are destroyed in lysosomes, and autophagy transports antigens destined for destruction and peptides from degraded antigens back to the cell surface for presentation on class II MHC (153).

B-lymphocytes are essential components of the immune system that are responsible for mounting immune reactions against infections and tumors by generating protective antibodies (173). Different B cell populations exhibit the ability to engage in both protective and destructive action (173). In addition to conventional facets of cellular metabolism, B cells rely on autophagy, a mechanism that facilitates the degradation of damaged cellular constituents (173). Autophagy is responsible for preserving metabolic balance in the absence of nutrients and promoting the extended survival of plasma cells (PCs) (174). Autophagy has been observed to facilitate the evasion of autoimmune checkpoints by self-reactive B cells, their activation through innate immune signals, and the presentation of autoantigens to T lymphocytes (175–177). Miller BC et al’s study elucidates the involvement of autophagy in B cell development by demonstrating that the introduction of Atg5-deficient cells into the fetal livers of Rag1–/– mice results in a developmental impediment at the pre-B cell stage (178). The findings suggest that the development of B2 cells requires autophagy, as evidenced by the unaffected splenic and lymph node B cell populations when Atg5 deletion was limited to mature B cells, while peripheral maintenance of these cells does not depend on autophagy (178). Autophagy was found to be a crucial process in the sustenance of mature B cell populations in the peripheral regions (179). Similar to other metabolic processes, autophagy plays a pivotal role in the functioning of B cells following activation. The study revealed that the absence of B cell autophagy did not affect the normal formation of memory cells two weeks post-immunization in mice. However, a significant reduction in the number of memory cells was observed after eight weeks, suggesting that autophagy is not a prerequisite for the formation of memory B cells, but plays a crucial role in their maintenance (180). Recent studies have demonstrated that AMPK regulates mitochondrial autophagy in memory B cells, thereby aiding in the mitigation of oxidative stress (181).

LC3-associated phagocytosis (LAP) is a newly discovered function of autophagy proteins that play a role in immune regulation and inflammation reactions in different cell and tissue types (182). LAP is a process that involves the conjugation of LC3 family proteins to phagosome membranes (182). It utilizes a segment of the conventional autophagy machinery, triggered by the binding of surface receptors that identify different types of cargos such as infectious agents, cell death, soluble ligands, and protein aggregates (182). Phagocytic cells may use LAP, in which the act of phagocytosis causes specific autophagy machinery to become activated and interact with the phagosome, facilitating its fusion with lysosomes (183, 184). LAP needs the same proteins as autophagy, including BECN1, VPS34-generated PI3P, Atg5, and Atg7; however, unlike autophagy, LC3 connects with the single phagosome membrane rather than the double membrane of autophagosomes (184). Additionally, in contrast to autophagy, the lysosomal acid phosphatase process does not require the first autophagy protein complex (ULK1, Atg13, and FIP200) instead, LAP involves a BECN1– VPS34 complex incorporating the Rubicon protein (184). Furthermore, LAP is an essential mechanism of immunosuppression in the microenvironment of tumors (184, 185).

Cytokines are a class of diminutive proteins that are secreted by cells and exert a distinct influence on intercellular interactions and communications (186). The presence of cytokines with pro-inflammatory and anti-inflammatory properties has been observed (186). The process of autophagy plays a critical role in regulating the synthesis and release of cytokines such as IL-1, IL-18, and TNF-α (187). Specifically, in human macrophages, cytokines such as IL-1, TNF-α, IL-6, IFN-γ, IL-2, and TGF-β have been shown to trigger autophagy, whereas cytokines like IL-4, IL-13, and IL-10 inhibit this process (154). Moreover, the release of cytokines due to the inflammatory response in the tumor microenvironment can lead to the recruitment and absorption of anti-tumor macrophages into the hypoxic region of the tumor (143).

IFN-γ is an essential cytokine that promotes inflammation that is mostly produced by natural killer cells and activated CD4+ or CD8+ T cells and innate and adaptive immunity depend heavily on IFN-γ (188, 189). New research reveals that IFN-γ increases autophagy, which in turn encourages antigen presentation, cellular growth, and the elimination of viruses and bacteria. In a process known as positive feedback, this autophagy activation then increases the release of IFN-γ (190, 191). Moreover, to assist eradicate invasive infections or causing cell death, IFN-γ may promote autophagy (192). Whereas the exact mechanism by which IFN-γ triggers autophagy is unclear, IFN-γ may activate macrophages through a pathway involving the family M member 1 GTPase Irgm1/IRGM1 (192). Autophagy can trigger the production of IFN-γ and promote the inflammatory response (193). As an illustration, conditional Atg5 knockdown significantly reduced IFN-γ induced LC3 conversion and autophagosome formation, which in turn reduced IFN-γ secretion by CD4+ T cells (192). Evidently, autophagy induced IFN-γ inducible inflammatory responses (194).

Endotoxins cause the creation of TNF-α, which hastens the development of several disorders linked to inflammatory responses (192). The idea that TNF-α and autophagy interact is supported by mounting evidence (195). Osteoclasts, epithelial cells, T lymphoblastic leukemic cells, skeletal muscle cells, and vascular smooth muscle cells are only a few of the cells that TNF-α primes for autophagy (195–200). It is unclear how TNF-α causes autophagy in various cell types (192). Depending on the cellular setting, autophagy either appears to up or down-regulate TNF-α production (192). One way that autophagy activation can reduce inflammatory reactions is by preventing the release of TNF-α (192). On the other side, autophagy has the potential to activate the inflammasome and cause the release of proinflammatory cytokines like TNF-α, IL-8, and IL-6 (192).

**Figure 4:**
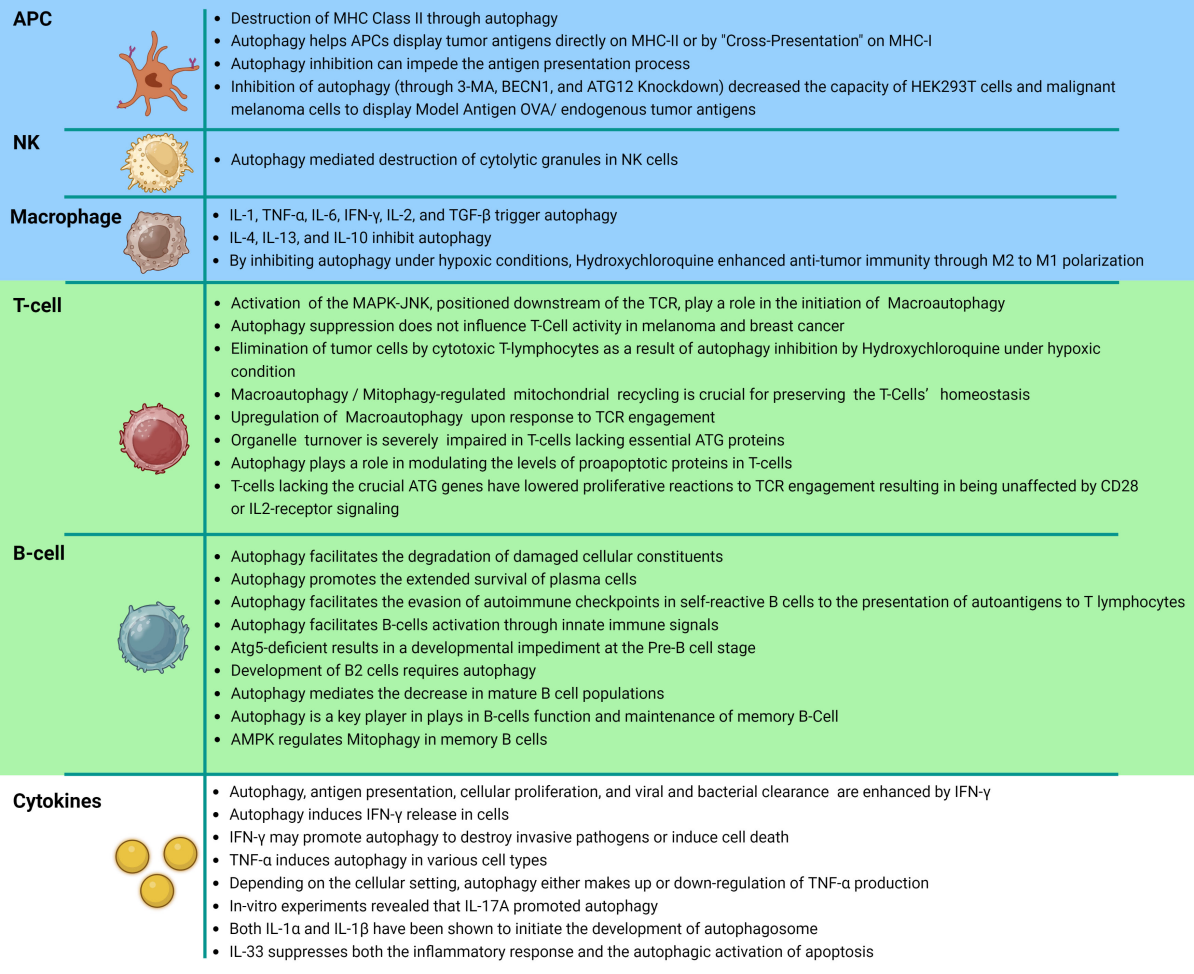
Autophagy related cancer immunity. This diagram represents a tabular structure, wherein the upper section with blue rows illustrates the interplay between innate immune cells, namely antigen-presenting cells, natural killer cells, and macrophages, in relation to autophagy. The central segment, highlighted in green, showcases additional interactions between the specific immune system and autophagy. It presents a detailed depiction and explanation of the involvement of T cells and subsequently B cells. Lastly, the lower section, depicted in white, elucidates the reciprocal interactions and consequential effects between cytokines secreted within the immune system and autophagy.

The inflammatory response is mediated by IL-17, which is largely produced by Th17 cells and plays a role in the formation of the tumor microenvironment (192). *In vitro* experiments on B cells revealed that IL-17A promoted autophagy (201). Recently, it was revealed that IL-1β can induce autophagosome formation in macrophages and epithelial cells in an inflammatory context, suggesting that IL-1β may induce autophagy as part of a negative feedback loop to reduce excessive inflammation and restore cellular homeostasis (202). This suggests that they may activate autophagy as a component of a negative feedback loop to reduce inflammation (202). According to a recent study by Gao Yung et al, IL-33 appears to suppress both the inflammatory response and the autophagic activation of apoptosis (203) (**Figure 4**).

Limited autophagy has cell-intrinsic implications, including genomic stress, DNA damage, and increased susceptibility to acquiring growth-promoting mutations (204, 205). Besides, autophagy can play a dual role in the cancer process, it regulates and prevents neoplasia, inflammation, and cancer; thus, these factors would be a common cancer trigger and produce a pro-tumorigenic environment (206–208). For instance, Crohn’s disease (CD) involves transmural inflammation of the terminal ileum (small intestine) but may affect the entire GI tract (209). Mouse hypomorphic for Atg16L1 or defective in Atg5 or Atg7 in the gut had significant cellular abnormalities localized to intestinal Paneth cells, similar to CD patients with the Atg16L1 risk gene (210, 211). Chronic intestinal and pancreatic inflammation, cancer risk factors, are characterized by impaired autophagy (29, 212).

As part of the cell’s reaction to xenobiotics, cytokines, and invasion by bacteria, as well as during mitochondrial oxidative metabolism, ROS are produced (213). ROS have been observed to be present at heightened levels in nearly all types of cancers, and are known to facilitate numerous facets of tumor growth and advancement (29, 212). Adequate levels of ROS are essential for the process of autophagy, and ROS accumulation in the tumor microenvironment hinders DC tumor defense action (29, 214). It is mostly derived from the respiratory chain of the mitochondria (29). ROS influences all phases of tumor development (212). Mitophagy is a process by which the cell gets rid of ROS-producing mitochondria that have been damaged (29). Mitophagy has two distinct molecular routes (29). When mitochondria are damaged, phosphatase and tensin homolog-induced kinase 1 recruit the E3 ubiquitin-like ligase PARKIN, which causes the voltage-dependent anion channel 1 (VDAC1) on the membrane of the mitochondria to get ubiquitinated and triggers the recruitment of p62 (215–217). Autophagic degradation is also aided by transferring oxidized proteins to p62 for destruction, and in many cancers, P62 is overexpressed (29, 218). Mathew et al. conducted a significant investigation that demonstrated the crucial requirement of p62 in tumorigenesis, revealing that depletion of p62 through autophagy suppressed tumor development (29, 219). Further molecular pathways supporting tumor growth were activated by persistent p62 expression that further changed nuclear factor-kappa B signaling (220). Oxidative DNA damage and increased tumorigenesis in autophagy-deficient cells are linked to p62’s inability to be properly cleared from the cell (29). Furthermore, mitochondrial BNIP3L may interact with Atg8 homologs and transport mitochondria to autophagosomes (95, 221).

Some studies suggest that it would be better to promote autophagy during cancer treatment to show the favorable effects (222). For instance, in breast cancer patients, the presence of increased LC3 puncta (indicative of autophagosomes) and nuclear HMGB1 correlated with improved overall survival, reduced metastasis, and enhanced tumor immune infiltration (223, 224). Interestingly, studies have demonstrated that suppression of autophagy does not affect T cell activity in preclinical models of melanoma and breast cancer, including cells treated with chemotherapy in the case of melanoma. However, within the hypoxic tumor microenvironment, autophagy upregulation in tumor cells inhibits immunological effector-induced cell death (143, 225). Nonetheless, under hypoxic conditions, treatment with hydroxychloroquine (HCQ) has been shown to improve T-cell death and enhance anti-tumor immunity by inducing a transition from an M2 to M1 polarization state in macrophages. This polarization transition facilitates the elimination of tumor cells by cytotoxic T lymphocytes (226, 227). Notably, despite the distinct mechanisms of action, both class I and II major histocompatibility complex (MHC) processing require activation of autophagy, even though a combination of starvation and rapamycin therapy induces autophagy, which reduces class II protein presentation (153, 228).

Inhibition of autophagy, either through 3-methyladenine treatment or knockdown of Beclin-1 or Atg12, significantly reduces the ability of human embryonic kidney cells (HEK293T) and malignant melanoma cells to present model antigen OVA or endogenous tumor antigens (229). Conversely, induction of autophagy yields the opposite effect (229). Autophagy plays a crucial role in antigen presentation by antigen-presenting cells (APCs) through a direct presentation on class II MHC or through “cross-presentation” on class I MHC (230). Pharmacological induction of autophagy with rapamycin in melanocytes significantly enhances the priming of CD8+ T cells by APCs presenting the melanocyte-derived tumor antigen gp100. Conversely, blocking autophagy with 3-methyladenine (3-MA) reverses this effect (229). Moreover, blocking autophagy by impairing autophagosome turnover increases antigen cross-presentation, indicating that autophagosomes serve as effective transporters of antigens from APCs to T cells. The stabilization of autophagosomes, rather than the initiation and completion of the autophagy process, is critical for cytotoxic T-cell priming (153, 229).

## 6 The role of autophagy in tumor suppression

There is evidence that autophagy plays a role in preventing tumorigenesis by several mechanisms. For example, autophagy can remove damaged organelles, particularly mitochondria that produce reactive oxygen species (ROS), which can damage cellular structures and promote genomic instability. Additionally, autophagy can promote the degradation of oncogenic proteins and inhibit their activity, thereby inhibiting their ability to promote carcinogenesis (24).

In cancer cell lines and mouse models, the absence of BECN1 has been shown to decrease autophagy and increase cell proliferation, providing evidence that BECN1 is a tumor suppressor gene (102, 103). Similarly, other proteins that interact with Beclin-1 and positively regulate autophagy, such as AMBRA1 (231), BIF-1 (110), and UVRAG (232), have demonstrated anti-proliferative or tumor-suppressive effects. Reported evidence indicate that AMBRA1 deficient mice models represent accelerated tumor growth, invasiveness, and metastasis in BRAF/PTEN melanoma phenotype via increased activity of Focal Adhesion Kinase 1 (FAK1) (231). UVRAG, as another Beclin1-binding protein, activates the Beclin1–PI3KC3 complex to promote autophagy and suppress the proliferation of human colon cancer cells (232). Furthermore, several studies have reported decreased levels of Beclin-1 in various types of cancer, including cervical squamous cell carcinomas and hepatocellular carcinomas (233–236). Consistent with the tumor suppressor hypothesis, the development of knockout mice for specific ATGs has revealed that deficiencies in certain regulators of autophagy are associated with a tumorigenic phenotype. However, because systemic deletion of Atg3, Atg5, Atg7, Atg9, or Atg16L1 results in neonatal mortality (12, 53, 237–239), the long-term effects of inhibiting autophagy could not be evaluated until mosaic Atg5 deletion mice were generated. In this context, mice with systemic mosaic Atg5 deletion or liver-specific deletion of Atg7 spontaneously develop benign hepatic adenomas (57).

**Figure 5:**
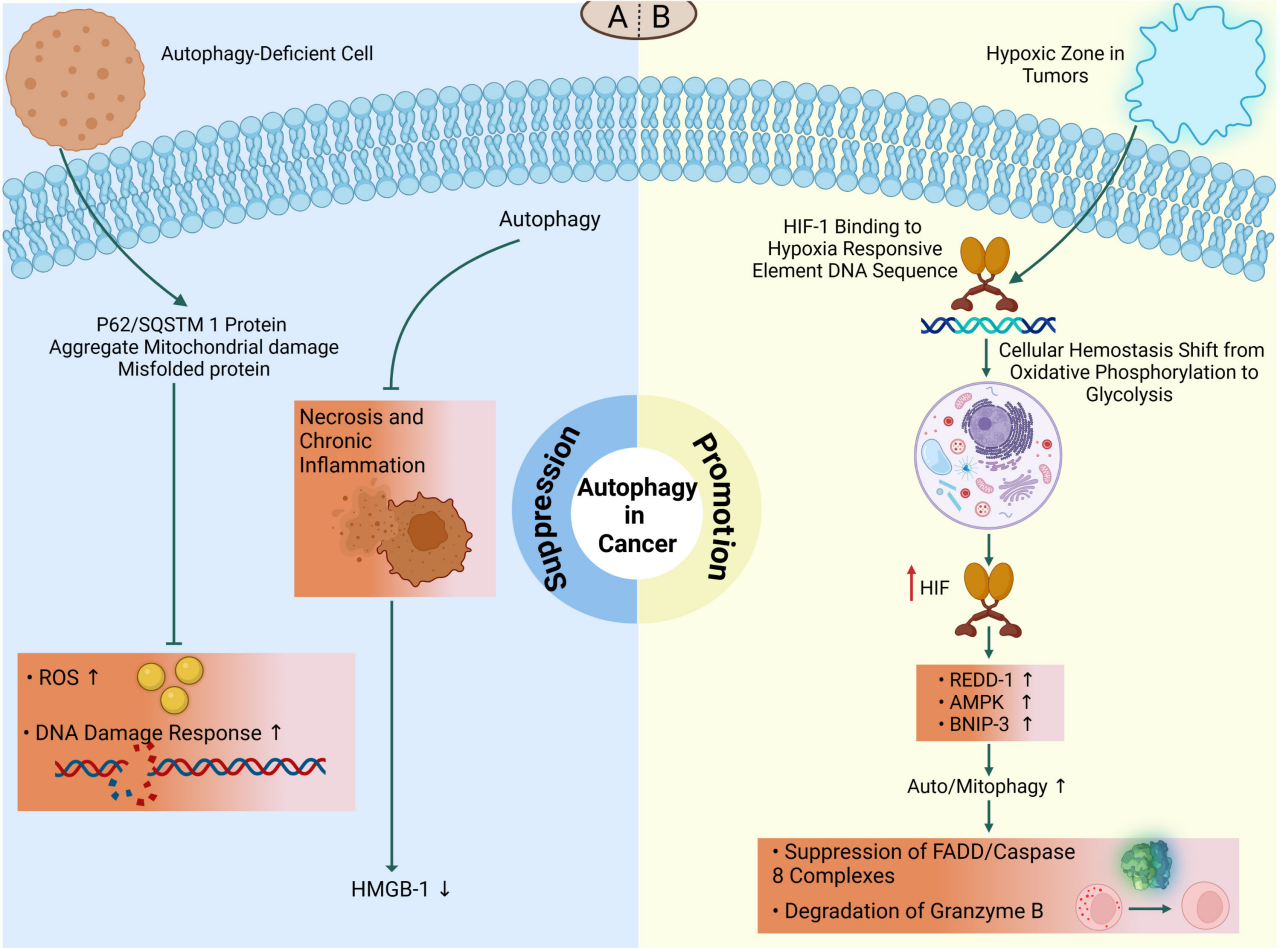
The dual role of autophagy in cancer. The double-edged nature of autophagy in tumor microenvironment demonstrates that it might function as a suppressor or promotor, depending on the type and stage of cancer. **(A)** On the one hand, autophagy suppresses tumor initiation and its deficiency may lead to tumorigenesis. Impaired autophagy shows increased DNA double-strand breaks and ROS production in defective cells via the accumulation of p62/SQSTM1 and other misfolded proteins which subsequently promotes tumorigenesis. Autophagy may also function in tumor suppression by mitigating tumor-associated necrosis and inflammatory response which is associated with release of HMGB1. **(B)** On the other hand, in hypoxic microenvironments, HIF-1 triggers hypoxia-induced autophagy to protect tumor cells from death. During this condition, HIF-1/HRE interactions contributes to oxidative phosphorylation to glycolysis transition which subsequently increases REDD1, AMPK, and BNIP3 to promote tumorigenesis via suppression of apoptosis-related factors (FADD/caspase-8 complex) or degradation of cellular lysing proteases (granzyme B).

Autophagy-deficient mice accumulate ubiquitinated keratins, the autophagy cargo adaptor p62, and aberrant mitochondria (12, 59, 219). Elevated levels of p62 and phospho-keratin 8 in several tissues and cancers, as well as in mammary tissues and malignancies, are potential biomarkers for autophagy defects (12, 240) aggregates or inclusions and are associated with forming ROS, activating the DNA damage response, cellular damage, and death. This can lead to chronic inflammation, promote degenerative and inflammatory disorders, and contribute to cancer development (9, 143, 210, 219). Chronic tissue injury and inflammation also contribute to the formation of DNA-damaging ROS, which can cause mutations and promote tumor growth (81). Deficient autophagy can lead to the development of p62/SQSTM1 protein aggregates, damaged mitochondria, and misfolded proteins, resulting in the formation of ROS that induces DNA damage and genomic instability (59). Knockdown of p62/SQSTM1 inhibited ROS and DNA damage responses in autophagy-deficient cells, indicating a potential molecular relationship between deficient autophagy and tumorigenesis (59). This association was also observed in p62/SQSTM1/mice, which were protected from Ras-induced lung carcinomas compared to wild-type animals (241). Autophagy may help protect against tumorigenesis by restricting necrosis and chronic inflammation connected with the production of pro-inflammatory HMGB1 (**Figure 5A**) (242). However, some results suggest a potential dual nature of this process in tumor development and progression. Overall, these findings suggest that autophagy can play a role in suppressing tumorigenesis in the early stages of cancer, but its effects can be complex and multifaceted.

## 7 The role of autophagy in tumor promotion

There is a substantial body of evidence indicating that autophagy plays a crucial role in promoting tumor development and survival, particularly in advanced malignancies (243, 244). This process is often triggered by hypoxic conditions within the central regions of solid tumors, which leads to the activation of autophagy. Deletion of the essential autophagy regulator Beclin-1 has been shown to suppress autophagy and increase cellular death (143, 245). Moreover, autophagy also supports the high metabolic and energy demands of rapidly growing malignancies by recycling intracellular components to provide essential metabolic substrates (24, 142).

Oxygen content is a critical metric influenced by the heterogeneity of tumors. Within the tumor, there are regions where oxygen content is less than 2%, creating a hypoxic zone (246). These hypoxic circumstances activate cellular pathways to maintain homeostasis. Hypoxia-inducible factor 1 (HIF-1) is the major transcriptional regulator under hypoxic settings. HIF-1 is a complex composed of two subunits, α and β. Under normoxic conditions (oxygen-rich), the α subunit degrades (247, 248). However, during hypoxia, the ubiquitylation of the α subunit decreases, leading to increased stability of HIF-1. HIF-1 binds to hypoxia-responsive element DNA sequences, which promotes a metabolic shift from oxidative phosphorylation (OXPHOS) to glycolysis (249). Regardless of oxygen concentration, HIF-1 upregulates the expression of approximately 80 genes essential for glucose metabolism, cell survival, tumor angiogenesis, invasion, and metastasis in tumor cells (249). HIF-1 increases AMPK in response to hypoxia or starvation, which further initiates autophagy through BINP3/Beclin-1 or mTOR suppression (250). In hypoxia, HIF-1 also promotes transcription of the regulated in development and DNA damage response 1 (REDD1), which activates the TSC1/2 complex, hence reducing mTOR activity and promoting autophagy (251). HIF-1 also increases the transcription of the gene encoding the Bcl-2/adenovirus E1 19kDa interacting protein 3 (BNIP3) that triggers mitophagy by releasing Beclin-1 from Bcl-2 family members, hence initiating autophagy (247) (**Figure 5B**). It has been demonstrated that hypoxia-induced autophagy may serve as a protective mechanism against apoptosis in hepatocellular carcinoma cells during periods of nutritional restriction, potentially through a Beclin-1-dependent pathway (252).

There are several reports suggesting the implications of autophagy as a preservative mechanism of tumor cells against immune responses. In a recent study, the protective role of autophagy in limiting T cell-mediated killing of tumor cells by TNF-α has been elucidated via suppression of FADD/caspase-8 complex activation (253). In addition, autophagy can limit the anti-tumor effect of NK cells in breast cancer cells via degradation of granzyme B in a hypoxic condition (**Figure 5B**) (254). Another mechanism of immune invasion is described in pancreatic cancer cells which involves degradation of MHC-I molecules in lysosomes to reduce the presentation of tumor cells following autophagy activation (255).

Autophagy is also enhanced in RAS-mutated cancer cells, which maintain a high basal-level of autophagy. RAS are small GTPases that participate in crucial signaling pathways for proliferation, survival, and metabolism (131, 256, 257). A mutation that activates RAS increases autophagy, which promotes tumor growth, survival, and oncogenesis, and is linked to the emergence of many lethal malignancies, including lung, colon, and pancreatic cancer (133–135, 258). Several studies have demonstrated that RAS-activating mutant cells exhibit an increased level of autophagy, which is essential for their survival during periods of nutrient deprivation (136, 259). Furthermore, inhibition of autophagy-related proteins leads to the accumulation of damaged mitochondria and a subsequent decrease in cell growth (141, 260, 261). Therefore, these findings demonstrate that autophagy plays a significant role in the survival of RAS-dependent tumor cells and can play a significant role in tumor promotion.

## 8 The role of autophagy in cancer metastasis

In addition to maintenance and differentiation of normal stem cells, autophagy is strongly correlated with cancer stem cell survival and maintenance, leading to tumor progression to secondary metastatic sites and therapy resistance.

Metastasis is influenced by several biological factors encompassing both intrinsic properties of cancer cells, including their migratory, invasive, and intracellular signaling abilities, as well as extrinsic properties of the microenvironment, such as the extracellular matrix (ECM) composition, interactions with other cell types, and vascular access, all of which collectively govern nutrient and oxygen availability (133). Given the many challenges that metastatic tumor cells must overcome in order to successfully establish distant colonies (such as invasion, anoikis resistance, and colonization) and the essential role of autophagy as a response to cellular stress, various roles for autophagy in the metastatic cascade have been hypothesized as mechanisms of tumor cell survival (39). Indeed, several environmental stresses known to promote metastasis, such as hypoxia, as well as those encountered by disseminated tumor cells, such as nutrition deprivation (262) and detachment from the ECM, activate autophagic flux (263–266). Currently, available data supports the notion that autophagy plays two distinct roles in cancer metastasis: either as a promotor or inhibitor, depending on the type of tumor cell, the tumor microenvironment and the steps of metastatic cascade (12, 13).

Despite the fact that we are currently unable to directly quantify autophagic flux in primary human tumor samples, several studies using surrogate markers have shown a relationship between enhanced autophagy and metastasis. Actually, autophagy-mediated metastasis promotion is achieved through two distinct mechanisms: 1. Disassembly of direct cell-to-cell contact 2. Enhanced secretion of pro-invasive factors. The dissemination and migratory abilities of tumor cell could be augmented by the detachment of adhesion molecules between tumor cells and migratory cells. Another important factor is the secretion of soluble components like interleukin 6 (IL-6), matrix metalloproteinase-2 (MMP2) and Wnt family member 5a (WNT5A) for induction of paracrine effects on recipient cells (267). These data suggest that autophagy is essential for invasive capacity of tumor cells.

Research on human breast and melanoma cancer revealed that higher light chain B (LC3B), as an autophagosome marker, punctate staining was associated with lymph node metastasis and decreased survival (268, 269). While melanoma metastases revealed enhanced LC3B staining as compared to original tumor samples with the same characteristics (268, 270, 271). LC3B expression has been discovered to be associated with metastasis in hepatocellular carcinoma. Specifically, increased LC3B staining has been observed in metastases compared to original tumors, as well as in early metastatic colonies compared to late metastatic colonies (272, 273). Increased expression of an autophagy gene signature was related with a more aggressive and invasive glioblastoma phenotype (274).

Numerous investigations have demonstrated an association between autophagy and epithelial-mesenchymal transition (EMT) in cancer, a process that facilitates the migratory capacity of tumor cells for invasion when their epithelial characteristics are lost for the acquisition of mesenchymal characteristics. During the metastatic phase, EMT-activated cancer cells have a high amount of autophagy to survive under several stressful situations (260, 261, 275). Another study showed that Cadherin-6, a type 2 cadherin that causes EMT during embryonic development, is abnormally increased in cancer and linked with cancer progression (276).

While autophagy promotes tumor growth and results in therapy resistance, new evidence has surprisingly demonstrated that autophagy suppresses the proliferation of disseminated tumor cells. According to one study, the activation of autophagy by nutrient deprivation and mTOR inhibition decreased glioblastoma cell migration and invasion. In addition, reduction of autophagy-related proteins, including Beclin-1, Atg5, and Atg7, enhanced the migration and invasion of glioblastoma cells with EMT regulators (277). It has been shown that *in vitro* and *in vivo* models of breast cancer metastasis could be inhibited through autophagy induction by CLDN6 as a component of actin cytoskeleton (278). Unexpectedly, a mammary cancer model experiment disclosed the contrary impacts of autophagy on cancer cells. While autophagy stimulates primary tumor expansion, metastatic growth is attenuated (279).

Collectively, these findings emphasize that due to the multi-faceted roles of autophagy during cancer progression, the prediction of overall outcome on tumor cells’ fate is dependent on the type of affected cell and the tumor stage, which requires to be evaluated on a context-dependent manner.

## 9 Therapeutic window

Cancer is a prevalent health concern on a global scale, accounting for approximately one-sixth of all fatalities across the world (280). The process of treating cancer has been characterized by a high degree of complexity (280). The treatment of cancer encompasses a diverse range of methods, such as surgical intervention, chemotherapy, radiation therapy, immunotherapy, and targeted therapy (280, 281). The selection of therapy is contingent upon the classification of neoplasm, its degree of advancement, and the general well-being of the individual (281). Tumors are frequently excised through surgical intervention, whereas cancer cells are targeted for destruction through chemotherapy and radiation therapy (281). Immunotherapy facilitates the recognition and eradication of cancerous cells by the immune system, whereas targeted therapy selectively targets molecules that are essential for the growth and survival of cancer cells (281). Combination therapies, comprising the utilization of multiple treatments, are frequently employed to attain optimal therapeutic results (281). As per the American Cancer Society, enhancements in the treatment of cancer have resulted in better survival rates and enhanced quality of life for individuals diagnosed with cancer (281).

The idea would have been that inhibiting autophagy in addition to conventional chemotherapy would be effective for treating tumors due to the survival benefits of autophagy that were originally shown *in vitro* (282). Nevertheless, several studies have shown that pharmacologically inhibiting autophagy using chloroquine may be advantageous for the treatment of tumors (283, 284). However, one can argue that promoting autophagy would be the best course of action when we take into account the potential implications this may have on a necrosis-driven pro-tumorigenic inflammatory response or the effects on preventing cellular senescence (282). According to prior research, two fundamentally distinct aspects of inhibiting or stimulating autophagy for the treatment of cancer are reviewed separately in this article.

Autophagy inhibition may be beneficial for cancer therapy, although also raises problems (285). Targeting alternative cell death pathways is an appealing method for enhancing anti-tumor therapy since apoptosis abnormalities are common in many solid tumor cells and can enhance tumor cell resistance to numerous traditional cancer therapies (286). Therefore, in cancer cells, the control of autophagy acts as a defense mechanism against chemotherapy (287). Autophagy inhibition in cancer therapy has drawbacks. For instance, given autophagy’s tumor-suppressive and protective effects in other systems (such as neurodegeneration, aging, and infectious illnesses), there are worries that inhibiting autophagy may increase the occurrence of secondary cancers or other disorders in treated individuals (154).On the other hand, the therapeutic benefits of chemotherapy and radiation are often counteracted by autophagy, which results in drug resistance (288). Restoring the vulnerability of tumor cells thus poses a prospective target for hematological malignancies (288). Autophagy decreases oxidative stress, inflammation, p62 accumulation, and genomic instability, which may suppress tumors in some model systems (285). Human cancer cells treated with HDAC inhibitors, arsenic trioxide, TNF-α, IFN- γ, rapamycin, and antiestrogen hormonal therapy activate autophagy as a pro-survival strategy, suggesting that inhibiting autophagy might make cancer cells more sensitive to these treatments (289–294). Autophagy serves as a cellular protection and defense mechanism that prevents cancer cell death during treatment, induces a state of dormancy in residual cancerous cells following treatment, promotes cancer-related recurrence and metastasis, and impedes cancer therapy and tumor cell eradication (283, 295).

It may not be possible to determine the side effects of systemic autophagy inhibition throughout the course of cancer treatment (154). However, recent research found that inhibiting autophagy might reduce chemotherapeutic responses by obstructing autophagy-dependent immune responses that fight cancer (6). Autophagy enhances tumor-cell survival under metabolic stress but also inhibits carcinogenesis, necrosis, and inflammation in one animal model when tumor cells have a deficiency in the apoptotic pathway, and autophagy regulation is a promising prospective method for improving cancer treatment (296–298). While the biological consequences on tumor cell behavior may change when the autophagy pathway is stopped at different stages of cancer, inducing autophagy may help prevent cancer since it suppresses tumors (222). What is more, Inhibiting autophagy and alkylating drugs enhance apoptosis and cell death (222).

Autophagy plays a cytoprotective or pro-survival role in cancer cells and can be induced by most cancer treatments including radiation therapy, chemotherapy, histone deacetylase inhibitors in colon cancer cells, arsenic trioxide (As2O3) in malignant glioma cells, Temozolomide (TMZ) in malignant glioma cells (299–306). Despite the fact that autophagy is upregulated in both tumor and normal cells exposed to cancer therapy, tumor cells rely more heavily on the cytoprotective benefits of autophagy than normal cells do (307). Certain chemotherapeutic drugs, such as etoposide, fenretinide, and dexamethasone, were discovered to induce autophagic cell death in cancer cells lacking critical apoptotic modulators such as BAX, BAK, or caspases *in vitro* (308–311). Both *in vivo* studies have shown that resveratrol and fisetin induce autophagic cell death in prostate cancer and Chronic Myelogenous Leukemia cell lines (312). Studies on malignant glioma cell lines, including U87MG and T98G, along with normal human astrocytes, have demonstrated that sodium selenite can selectively induce mitophagic cell death in glioma cells while sparing normal astrocytes. These observations signal that sodium selenite could become a promising therapeutic strategy for glioblastoma, presenting a mitochondria-selective way of inducing autophagic cell death (313, 314).

As a synthetic guanidine derivative, metformin is used to treat the symptoms of diabetes (287). By activating autophagy in cancer cell lines and animal models, metformin also has anticancer effects (287). In endometrial cancer cells, metformin produces cell cycle arrest, which prevents cell viability and proliferation, and promotes apoptosis by triggering autophagy (315). Additionally, metformin induces the autophagic flow by increasing the levels of LC3-II and decreasing the levels of p62, which leads to TRAIL-mediated apoptosis in TRAIL-resistant lung cancer cells (316).

In this section of the article, we have discussed various therapeutic issues in the field of autophagy. We have pointed out the importance of cancer prevention by promoting autophagy. In addition, we have expanded the discussion about the effect of autophagy inhibition on anticancer therapy. Moreover, we have investigated the signaling pathways related to the initiation and inhibition of autophagy. In addition, we have written a detailed discussion on the suppression of autophagy by chloroquine (CQ) and hydroxychloroquine (HCQ). Finally, we have introduced a future prospective view of new medications.

### 9.1 Autophagy in precision medicine

The fact that approximately 70% of clinical studies are dedicated to exploring the role of autophagy in cancer indicates the promising potential of modulating autophagy for cancer treatment. Clinical trials have been designed to investigate the impact of autophagy modulation in combination with conventional therapies (317). Thirty-six genes involved in the autophagy pathway are associated with the risk, diagnosis, and clinical outcome of 30 different types of cancer (318). Therefore, a potentially effective strategy for treating cancer could involve targeting autophagy through a combination of autophagy modulators and chemotherapeutic agents.

Polymorphisms in autophagy genes, including certain ATG SNPs, predispose individuals to develop a wide variety of diseases such as cancer. Notably, PIK3C3 SNPs are more frequently found in different gastrointestinal cancers (319, 320). Similarly, variations in all components of the ATG12 conjugation system are linked to different solid tumors (321–323), highlighting the significance of autophagy in the development of cancer.

Various clinical trials are testing the efficacy of anticancer therapies utilizing autophagy modulators. PI3K and MTOR inhibitors have been implemented in hematological malignancies such as CLL and T-ALL, as well as hepatocellular carcinoma (324–326). Moreover, the interplay between autophagy and apoptosis plays a significant role in multiple myeloma (MM) progression and drug resistance. A potential approach to combat MM cell survival may include inhibiting autophagy to trigger apoptosis (327).

However, predicting the therapeutic response of diseases to autophagy alteration is context-dependent and whether autophagy acts as a tumor suppressor or an oncogenic factor. It is crucial to determine precisely when to regulate to achieve optimal effects as an adjuvant therapy for tumors. Therefore, to achieve more effective personalized treatment responses, further investigation and research into strategies for activating or inhibiting autophagy are necessary under different conditions.

### 9.2 Autophagy and cancer prevention: mechanistic insights and future prospects

Currently, preventive treatments are important in the management of cancer, including its hereditary types. The development of endocrine resistance poses a significant challenge in the management of breast cancer that is positive for estrogen receptor expression (328). The relationship between autophagy and endocrine resistance is not yet fully understood, despite the growing attention given to autophagy as a potential contributing factor (328). The administration of Tamoxifen (Tam) induces autophagy and influences the lysosomal compartment of MCF7 cells (328). This results in the activation of autophagy, which facilitates the elimination of Tamoxifen-damaged lysosomes through lysophagy (287, 328, 329). The MCF7-TamR cells, which are resistant to 5 µM tamoxifen, exhibit an increased autophagic flux and greater resistance to Tam-induced lysosomal alterations in comparison to the parental cells (328). This indicates a potential correlation between these two phenomena (328). Autophagy inhibition re-sensitizes MCF7-TamR cells, which overexpress metallothionein 2A and ferritin heavy chain mRNAs (328). In parental MCF7 cells, overexpressing these proteins protects lysosomes against Tam-induced damage and retains survival, but suppressing autophagy removes protection (328). Chiara Actisshow and colleagues showed that additional breast cancer cells that overexpress certain iron-binding protein mRNAs are less vulnerable to Tam-induced lysosomal degradation when autophagy is initiated (328).

Nonetheless, it should be noted that there exists solely a correlative association between the decrease in cancer incidence and the implementation of autophagy-promoting strategies (154). If autophagy upregulation has a mechanistic role in the effectiveness of such cancer prevention measures, further research is needed to confirm this (154). If true, using more direct autophagy activators could provide a workable new alternative cancer prevention technique (154).

### 9.3 Autophagy inhibition as an adjunctive therapy for anticancer treatment: current status and emerging strategies

Anticancer therapies are made more effective in a variety of cancer cells by suppressing autophagy via genetic or pharmacological means (330–332). Different autophagy inhibitors may be used alone or in conjunction with other anticancer medications for the treatment of cancer. In this regard, by increasing caspase activity and decreasing cell survival, the suppression of autophagy by Atg5 and Beclin-1 siRNA improves cisplatin sensitivity in lung cancer cells (333). Similarly, 3-methyladenine (3-MA), an autophagy inhibitor, promotes hypoxia-induced apoptosis in colorectal cancer by suppressing autophagy (334). In addition, the effectiveness of enzalutamide (ENZ) in bladder cancer is limited by the resistance that is induced by an increase in AMPK, Atg5, LC3B, and ULK1 levels due to the stimulation of autophagy (335).

In many cancer cells, the cholesterol-lowering drug atorvastatin (ATO) exhibits anticancer effects (287). The stimulation of apoptosis-related proteins such as caspase-3, PARP, and Bim by ATO reduces cancer development and increases apoptosis in cervical cancer cells (287). Autophagy is induced as a side effect of ATO therapy, which limits the therapeutic impact of cancer medications. Combining ATO with an autophagy inhibitor, such as 3-MA or Bafilomycin A1 (Baf A1), enhances ATO-induced apoptosis in cervical cancer cells (336). Reactivation of p53 and induction of tumor cell apoptosis (RITA) is a small chemical that disrupts the link between p53 and mouse double-minute 2 homolog (MDM2) and exhibits anticancer benefits by exclusively causing apoptosis, however, resistance to the drug is a significant obstacle in the treatment of cancer (287). Through the suppression of autophagy and promotion of apoptosis, the combination therapy with RITA and 3-MA has excellent therapeutic effects on cisplatin- and RITA-resistant head and neck cancer cells (337).

### 9.4 Baf A1

Baf A1 demonstrates autophagy suppression and augmentation of apoptosis (287). However, the therapeutic benefit of Baf A1 is only seen at high doses, and its use is restricted owing to the possibility of toxicity (338). Baf A1 inhibits autophagy, targets mitochondria, and induces apoptosis to have therapeutic benefits in juvenile B-cell acute lymphoblastic leukemia (ALL) at low doses (339). In bladder cancer, cisplatin causes autophagy to become activated, which then results in cisplatin resistance (340). By preventing autophagy, Baf A1 therapy enhances the therapeutic effects of cisplatin (287). Because autophagy plays a role in the development of 5-FU resistance in gastric cancer, treating the disease with Baf A1 in combination reduces autophagy, which suppresses autophagy and reduces the capacity of cancer to clone, invades, or migrates (341). Another study found that in BRAF-mutant melanoma, cytoprotective autophagy plays a key role in the development of resistance to BRAF inhibition (342). This result was particularly noteworthy since, despite extensive research on the role autophagy plays in PI3K/AKT/mTOR signaling resistance to targeted therapeutics, the role of autophagy in MAPK pathway suppression has received less attention (343, 344).

### 9.5 PI3K-AKT-mTOR signaling

Signaling pathways are very important in understanding a biological phenomenon, according to changes in these pathways, a disease may start or cure. Today, the main basis of some treatments is based on major or minor changes in biological signaling pathways. A prospective chemotherapeutic target that is often activated in various tumors is the PI3K/mTOR pathway (345, 346). PI3K-AKT-mTOR signaling is dysregulated in human cancers, and mTOR inhibition induces autophagy (347). In a mouse model, treatment with mTOR inhibitor rapamycin was connected to a 90% decrease in lung cancers brought on by carcinogens (348). Similarly, in the same tumor model, metformin’s inhibition of mTOR signaling reduced tumorigenesis (349). In addition, ongoing low-dose rapamycin therapy significantly reduced intestinal neoplasia in APCMin/+ mice with increased AKT-mTOR signaling (350).

Through a range of signaling pathways, including the DNA damage response, mTOR and AMP-activated protein kinase signaling, the ER stress response, and others, traditional cytotoxic chemotherapeutics and tailored treatments promote autophagy (351). Unc-51-like kinase 1 (ULK-1) is a small molecule inhibitor of ULK1 that, in response to various stimuli, suppresses both autophagy and autophagy flux (352). There are only two serine/threonine kinases in the autophagy pathway, ULK1, and ULK2, which makes them a great target for therapeutic intervention (353). Inhibitors of ULK1 kinase that compete with ATP include the selective SBI-0206965 (SBI) (353). The combination of SBI and mTOR suppresses and controls autophagy and works in concert with other common chemotherapies, such as mTOR inhibition (353, 354). The mTORC1 inhibitors temsirolimus and everolimus, promote autophagy (18).

SAR405 is a PIK3C3 low molecular mass kinase inhibitor that prevents autophagy by reducing PIK3C3’s catalytic activity (355). SB02024 was demonstrated to successfully inhibit autophagy, decrease breast cancer xenograft models, and work in synergy with other treatments *in vitro* (356). VPS34 inhibitors have been created, including SB02024 and VPS34-IN1 (357). Inhibitors of ULK1 and VPS34 have also been demonstrated to be efficient in CNS tumor cells that depend on autophagy (358).

To determine autophagy reliance, epidermal growth factor receptor (EGFR) mutations or amplifications have also been used (222). Numerous downstream pathways, including PI3K-AKT-mTOR, STAT3, and RAS family signaling, are regulated by EGFR and have an impact on autophagy (359). Radio resistance is linked to EGFR mutations and amplifications in head and neck squamous cell carcinomas and glioblastoma (GBM) (222). These cancers respond to pharmacological inhibition of autophagy and are highly autophagy-dependent (360).

The least Drugs including rapamycin analogs (mTOR inhibitors), class I PI3K inhibitors, and metformin (an AMPK activator) have been shown to suppress malignant transformation when they pharmacologically activate autophagy (58, 361, 362). Additionally, Atg7 inhibitors might theoretically also be used to target the conjugation machinery (222). It has been demonstrated that Atg7 can be genetically targeted to stop this enzyme in cancer cells (363, 364).

With therapeutic effects on leukemic cell lines and B-cells generated from patients through inhibition of the PI3K/AKT pathway and activation of autophagy, quercetin is a natural flavonol and a multi-kinase inhibitor that restores the sensitivity to ABT-737 (365). ABT-737 and its derivatives (ABT-263 and ABT199) inhibit the interaction between Beclin-1 and Bcl2 to induce autophagic-like cell death, which has anticancer effects on glioblastoma cells (366). As a possible ALK/IGF1R inhibitor, AZD3463 demonstrates an anticancer impact and, via controlling the PI3K/AKT/mTOR pathway, triggers apoptosis and autophagy (367). Through the activation of apoptosis, autophagy, and a decrease in cell proliferation in breast cancer cells, the co-treatment of AZD3463 with rapamycin boosts the effectiveness of anticancer therapy (368).

The flavonoid isoliquiritigenin (ISL), which is produced from Glycyrrhiza glabra, has anticancer properties both *in vivo* and *in vitro* (287). By altering the PI3K/AKT/mTOR pathway, ISL causes the suppression of cell development by boosting apoptosis and autophagy (287). By promoting ISL-mediated apoptosis, the autophagy inhibitor HCQ enhances the therapeutic impact of anticancer treatment against HCC (367).

### 9.6 Chloroquine and hydroxychloroquine

Autophagy was directly suppressed by chloroquine (CQ) and hydroxychloroquine (HCQ), which have previously been used to prevent and cure malaria (369), by altering lysosomal pH, stopping autophagic breakdown, and increasing autophagosomal accumulation (370, 371). The autophagy inhibitors CQ and HCQ are utilized in conjunction with various targeted regulators or chemotherapeutics, such as bortezomib and cyclophosphamide (372). Temsirolimus (CQ: antimalaria agent), everolimus (HCQ: CQ derivative), and hydroxychloroquine (HCQ: CQ derivative) are some of the autophagy regulators and apoptosis triggers utilized in cancer treatment including bladder cancer (369, 373). Similar to CQ, HCQ is a weak basic tertiary amine that may build up in the lysosome’s acidic environment, where it is protonated and prevents lysosome diffusion (374, 375). The lysosome’s pH rises consequently, inhibiting lysosomal activity and autophagy in the process (375). Combining CQ or HCQ with metabolic stresses (such as an angiogenesis inhibitor or 2-deoxyglucose) or targeted therapeutic medications (such as imatinib, a Bcr-Abl inhibitor) has also been shown to increase the risk of cell mortality (376, 377). CQ and HCQ have decreased cancer cell proliferation in the bladder and pancreatic adenocarcinoma preclinical tests (369, 373).

Arsenic trioxide is one example of a common medication that causes autophagy rather than cell survival (372). Therefore, when autophagy acts as a pro-survival mechanism in response to a particular treatment strategy, autophagy inhibitors should be utilized (288).

CQ’s inhibition of autophagy in preclinical models enhances tumor cells’ reactivity to alkylating drugs, indicating that autophagy aids in survival (284). Autophagy induction is similar between HCQ and CQ, despite the fact that HCQ is less toxic (378). Combination therapy with ENZ and CQ enhances the therapeutic efficiency by decreasing tumor growth and inducing apoptosis, which is achieved by genetic suppression of autophagy using Atg5 siRNA (335).

It has also been shown that CQ may increase the apoptosis and the therapeutic benefits of PS-PDT in colorectal cancer cells via inhibiting autophagy (379). CQ and HCQ suppress autophagy by reducing autophagosome/lysosome fusion (380). CQ also has autophagy-independent anti-cancer properties and can sensitize cancer cells to treatment (381–383). Patients’ clinical results were noticeably improved in the first clinical study of CQ for the treatment of GBM patients (384).

Autophagy inhibition changes normal and malignant cell responses to other therapies, hence the maximum tolerable dose (MTD) of HCQ varies with concurrent therapy (222). In studies with HCQ with targeted treatments like vorinostat, the MTD was 600 mg twice daily, whereas, with cytotoxic chemotherapy like temozolomide and radiation in GBM, the MTD was 400 mg twice daily (385). As a single agent, HCQ’s MTD is unknown, and 600 mg twice daily in adults is the greatest dosage studied to date, however, autophagy inhibition was not consistently attained at that level (385).

Pharmacokinetic investigations in dog lymphoma patients utilizing HCQ and doxorubicin showed a 100-fold increase in tumor HCQ compared to plasma concentrations, suggesting a divergence between tumor exposures and evaluating autophagy inhibition in PBMCs as a proxy for efficacy (381).

Examples of completed research include a Phase I study that combined HCQ with bortezomib, a proteasome inhibitor, in patients with relapsed or refractory multiple myeloma, and found that the combination had a better impact than bortezomib alone in the past (386, 387). Pancreatic ductal adenocarcinoma (PDAC) patients who had progressed through multiple lines of therapy and were given only HCQ as monotherapy in a Phase II trial showed no objective responses, but it’s possible that this was due to the fact that these patients were heavily pretreated population and didn’t receive HCQ for a long enough duration to achieve therapeutic doses of the drug (388). Preoperative HCQ and gemcitabine treatment exhibited responsiveness to the CA19-9 tumor marker and improved overall survival when compared to historical controls in Phase I and II trials of HCQ-mediated autophagy suppression in PDAC (389).

In a phase I clinical study, HCQ and chemotherapy drugs together improved 18 patients with relapsed or refractory multiple myeloma’s median progression-free survival (mPFS) and overall survival (OS) (390). A study was conducted to evaluate the therapeutic efficacy of combining HCQ with the histone deacetylase inhibitor vorinostat (VOR) in 19 patients diagnosed with metastatic colorectal cancer (391). In patients with refractory colorectal cancer, the combination therapy demonstrated 2.8 months progression-free survival (mPFS) and 6.7 months OS, confirming its safety and well-tolerance in these patients (287, 391).

Thirty-five patients with borderline resectable pancreatic adenocarcinomas were treated in a phase 1/2 experiment with doses of fixed-dose gemcitabine (1500mg/m2) and HCQ at a dosage of 1200 mg per day until the day of surgery (389). The experiment proved safe and well-tolerated pre-operative autophagy suppression with HCQ with gemcitabine (389). The fact that 29 out of 35 patients received surgical resection and 19 out of 35 patients demonstrated a drop in surrogate biomarker response suggests autophagy inhibition with HCQ might have a beneficial effect (389).

Everolimus, also known as RAD-001, is a rapamycin derivative (287). In endometrial cancer and HEC01A cells, RAD-001 increases susceptibility to paclitaxel-induced apoptosis by inducing autophagy through the downregulation of AKT/mTOR phosphorylation and accumulation of LC3 (392). In pancreatic cancer PC-2 cells, rapamycin generates autophagic vacuoles, which inhibit proliferation and triggers death (393). It also increases the expression of Beclin-1 in a dose-dependent way (393). Everolimus was evaluated in phase I clinical study on women who had lymphangioleiomyomatosis, together with the autophagic flux inhibitor HCQ (287). Patients with advanced cancer have undergone testing combining rapamune (also known as rapamycin; commercial name) with HCQ (287).

It is crucial to highlight that *in vivo* genetic proof that the effects of CQ in these conditions were via regulation of autophagy has yet to be demonstrated (222). CQ’s effects, however, are not confined to autophagy suppression, causing, among other things, inhibition of lysosomal activity generally (222). In this context, it is noteworthy that a separate investigation into the therapeutic efficacy of CQ found prodeath consequences of autophagy (222). In this situation, the cell death brought on by CQ treatment was decreased by caspase inhibition in an autophagy-deficient background but not in autophagy-competent cells (222). This would suggest that autophagy is somewhat required for cell death downstream of CQ in this environment (222). In **Table 1**, the clinical trials investigating the effects of chloroquine and hydroxychloroquine on various cancers are presented.

**Table 1:** Clinical trials with Chloroquine (CQ) and hydroxychloroquine (HCQ) for Cancer treatment.

Reference	Phase	Drug combination	Cancer type
**NCT02071537**	Phase I	CQ/HCQ + Carboplatin Gemcitabine	Malignant Neoplasm Solid Tumors
**NCT01727531**	Phase II	CQ	Brain Metastasis
**NCT01777477**	Phase I	CQ + gemcitabine	Pancreatic Cancer
**NCT01023477**	Phase I/II	Chloroquine at Low Dose	Ductal Carcinoma *in situ* Breast Cancer
**NCT00224978**	Phase III	None	Glioblastoma Multiforme
**NCT02496741**	Phase I/II	CQ + Metformin	Glioma Cholangiocarcinoma Chondrosarcoma
**NCT02378532**	Phase I	CQ + chemoradiation with temozolomide	Glioblastoma Multiforme
**NCT01446016**	Phase II	CQ + Taxols	Breast Cancer
**NCT02333890**	Phase II	CQ	Breast Cancer Invasive Breast Cancer
**NCT04772846**	Phase I/II	CQ	Glioblastoma
**NCT02366884**	Phase II	CQ + Anti-Bacterial Agents/Anti-Fungal/Agent Anti-Protozoal Agents	Malignant disease
**NCT03400865**	Unknown	CQ	Prolactinoma
**NCT01023737**	Phase I	HCQ + Vorinostat	Malignant Solid Tumour
**NCT01266057**	Phase I	HCQ + Sirolimus Vorinostat/Sirolimus	Advanced Cancers
**NCT03015324**	Phase I	HCQ	Solid Tumor
**NCT01273805**	Phase I	HCQ	Pancreatic Cancer
**NCT04145297**	Phase I	HCQ + Ulixertinib	Gastrointestinal Neoplasms
**NCT03377179**	Phase II	HCQ + ABC294640	Cholangiocarcinoma
**NCT01006369**	Phase II	HCQ + Bevacizumab + XELOX	Colorectal Cancer
**NCT02232243**	Phase I	Low dose HCQ	Solid Tumor
**NCT00726596**	Phase II	HCQ	Prostate Cancer
**NCT00568880**	Phase I	HCQ + Bortezomib	Multiple Myeloma
**NCT01634893**	Phase I	HCQ + Sorafenib	Solid Tumors
**NCT01128296**	Phase I/II	HCQ + Gemcitabine	Pancreatic Cancer
**NCT04386057**	Phase II	HCQ + LY3214996	Pancreatic Cancer
**NCT03215264**	Phase I	HCQ/Entinostat/Regorafenib	Colorectal Cancer
**NCT00962845**	Phase I	HCQ	Melanoma
**NCT03081702**	Phase I/II	HCQ + Itraconazole	Ovarian Cancer
**NCT02316340**	Phase II	HCQ + Vorinostat	Colorectal Cancer
**NCT03513211**	Phase I/II	SUBA-itraconazole	Prostate Cancer
**NCT01978184**	Phase II	HCQ + Gemcitabine + Nab Paclitacel	Pancreatic Cancer
**NCT00486603**	Phase I/II	HCQ + Temozolomide + Radiation therapy	BrainTumors Central Nervous System Tumors
**NCT03774472**	Phase I/II	HCQ + Letrozole + Palbociclib	Breast Cancer
**NCT04593758**	Phase I/II	HCQ + CPI-613	Melanoma
**NCT00813423**	Phase I	HCQ + Sunitinib Malate	Solid Neoplasm
**NCT01550367**	Phase I/II	HCQ + Aldesleukin	Metastatic Renal Cell Carcinoma
**NCT01649947**	Phase II	HCQ	Non-small Cell Lung Cancer
**NCT01602588**	Phase II	HCQ + Short course radiotherapy	Glioblastoma
**NCT01396200**	Phase I	HCQ + Cyclophosphamide and Pulse Dexamethasone	Myeloma
**NCT01510119**	Phase I/II	HCQ + RAD001	Metastatic Clear Cell Renal Cell Carcinoma
**NCT01897116**	Phase I	HCQ + Vemurafenib	Melanoma
**NCT01506973**	Phase I/II	HCQ + Gemcitabine/Abraxane	Pancreatic Cancer
**NCT04163107**	Phase I	HCQ + Carfilzomib + Dexamethasone	Multiple Myeloma
**NCT01206530**	Phase I/II	HCQ + FOLFOX + Bevacizumab	Adenocarcinoma Rectal Cancer Colon Cancer
**NCT00909831**	Phase I	HCQ + Temsirolimus	Solid Tumor
**NCT02257424**	Phase I/II	HCQ+ Trametinib + Dabrafenib	Melanoma
**NCT01689987**	Phase I	HCQ + Cyclophosphamid + Dexamethasone + Sirolimus	Multiple Myeloma
**NCT00809237**	Phase I/II	HCQ + Gefitinib	Non-small Cell Lung Cancer
**NCT04341207**	Phase II	HCQ + Azithromycin	Possibility of anticancer immune responses
**NCT00977470**	Phase II	HCQ + Erlotinib	Non-small Cell Lung Cancer
**NCT01227135**	Phase II	HCQ + Imatinib mesylate	Chronic Myeloid Leukemia
**NCT03754179**	Phase I/II	HCQ + Dabrafenib + Trametinib	Melanoma
**NCT01494155**	Phase II	HCQ + Short course radiotherapy + Capecitabine	Pancreatic Cancer
**NCT01292408**	Phase II	HCQ	Breast Cancer
**NCT03979651**	N/A	HCQ +Trametinib	Metastatic NRAS Melanoma

### 9.7 New medications

Next-generation lysosomal targeted inhibitors that are more effective and selective are now being developed, including Lys05, a bisaminoquinoline, and DQ661, a dimeric quinacrine with the added advantage of concurrent lysosome and mTOR inhibition (394). Lys05, which is around ten times more powerful than CQ, has shown effective in controlling the formation of colorectal adenocarcinoma and melanoma in animal models Due to a larger concentration in and deacidification of the lysosome, Lys05 is a more powerful autophagy inhibitor than HCQ (395). CQ inhibits autophagy by preventing autophagosome-lysosome fusion, unlike Lys05 (380). In melanoma and colon cancer xenograft models, Lys05 demonstrated more anticancer effects than HCQ both *in vitro* and *in vivo* (381, 395).

Additionally, DQ661 has demonstrated significant *in vivo* single-agent effectiveness against melanoma and colorectal cancer, as well as *in vivo* efficacy against PDAC when combined with gemcitabine as a single treatment, HCQ has demonstrated very modest clinical responses in cancers such as advanced metastatic pancreatic cancer (394).

There exist supplementary techniques to modulate autophagy, such as the utilization of epigenetic modifiers (222). The regulation of autophagy via epigenetic mechanisms has been evidenced by the acetylation of histones, hyper-methylation of CpG islands, and interference with mRNA activity caused by noncoding RNAs in the cytoplasm (396). Histone deacetylase inhibitors used to treat malignant nerve sheath tumors increased autophagy and therapy resistance (397). Consequently, the search for additional CQ analogs like Lys05 and medicines that affect other parts of the autophagic pathway is at an all-time high (398).

Cancer cells may rely on noncanonical mechanisms such as LC3-associated phagocytosis that have been overlooked in the rush to target the canonical autophagic route (399). Some dietary phytochemicals, including quercetin, apigenin, genstein, hesperein, and luteolin have recently been proven to trigger autophagy in normal cells as well as numerous types of cancer cells (400, 401).

Autophagy addiction, also known as autophagy-dependence, is essential because only autophagy-dependent tumors react strongly to autophagy inhibition, even when the same medicines with CQ are tried (402). The effect of other drugs and autophagy inhibitors is additive in autophagy-dependent tumor cells, while these drugs have an antagonistic effect on autophagy-independent tumor cells (402, 403).

BrafV600E-driven lung and melanoma mouse models were among the first to show autophagy dependency and sensitivity to Atg7 deletion (363, 404). Following clinical studies, PDAC patients treated with gemcitabine, nab-paclitaxel, and HCQ had better surgical results (389, 405). Recent studies showed the advantage of inhibiting autophagy together with MEK or ERK to target the RAS pathway (406, 407). Multiple cancer cell lines have been treated with recombinant Bacillus caldovelox arginase mutant (BCA-M), which is effective in anticancer treatment by slowing the proliferation of human cervical cancer cells (408). Through the reduction of growth, an increase in apoptosis, and cell cycle arrest, BCA-M demonstrated beneficial therapeutic effects on cancer cells in phase III clinical study (287). Additionally, by lowering autophagy, combined therapy with BCA-M and CQ enhances the therapeutic benefits of BCA-M (287).

Class III PI3K kinase complexes are removed by proteasomal degradation when Spautin-1, a protein that normally suppresses autophagy, is present (409). Spautin-1’s ability to promote apoptosis is linked to GSK3β, a key downstream effector of PI3K/AKT (409).

SAR405 also inhibits Vps18 and Vps34 kinases, impairing lysosomal function and interfering with the late endosome-lysosome interface (410). The combination of SAR405 with everolimus has been shown to increase the suppression of kidney cancer cell growth (410). Furthermore, Vps34 inhibitor SAR405 has anticancer therapeutic benefits, as shown by these data (18). Everolimus and SAR405 therapy combined results in a synergistic anticancer impact by reducing kidney cancer cell growth (410). By inhibiting autophagy by specifically targeting Vps34, SB02024, a novel, and highly effective selective inhibitor, increases sensitivity to sunitinib and erlotinib (356).

Doxorubicin is a DNA-damaging drug that causes aberrant mitochondrial activity and the creation of superoxide to have an anticancer impact (411). Doxorubicin sensitivity is restored in colorectal cancer stem cells by inhibiting mitophagy by silencing BNIP3L, a key regulator of mitophagy (412).

Foxk proteins (Foxk1 and Foxk2) functioning as transcriptional repressors of autophagy genes have been used to illustrate the transcriptional control of autophagy (413, 414). In nutrient-rich environments, mTOR stimulates Foxk1’s transcriptional activity, which leads to Foxk1 and Sin3A co-localizing at the promoters of 79 known autophagy-associated genes (413). Intriguingly, Foxk1 siRNA ablation led to the overexpression of essential Ulk1 and Vps34 machinery, highlighting the detrimental effect Foxk1 transcriptional activity has on autophagy (414). Studies, that starvation triggers a transcriptional mechanism predominantly controlled by the transcription factor EB (TFEB), which results in the activation of autophagy and lysosomal genes to help the cell survive, autophagy has been connected to lysosomal biogenesis (415). TFEB, when overexpressed, greatly increases the number of autophagosomes in cells (413).

The mice models outlined for examining the potential impact of mutant p53 on the susceptibility to autophagy-based treatment have been supplemented by the discovery that P53 plays a function in the transcription of autophagy genes (413). Global genomic profiling of mouse embryo fibroblasts showed p53 to transcriptionally control a large number of autophagy genes, with p53 transcriptional activity being required for the activation of autophagy in response to DNA damage (416). It is important to note that autophagy has been shown to continue even in the absence of functioning p53, indicating that p53 plays a role in the intricately choreographed symphony that is autophagy rather than just regulating it (417).

In autophagic cells, the epigenetically determined acetylation state of histone H4 lysine 16 (H4K16) controls choices about life and death (418). When autophagy is induced, H4K16 acetylation (H4K16ac) declines, which in turn reduces the expression of ATG genes throughout the whole genome (418). An increase in autophagic cell death is caused by reversing the decrease in H4K16ac that occurs when autophagy is induced (418).

Mitophagy is reduced by mitochondrial division inhibitor 1, or mdivi-1, a specific inhibitor of dynamin I and the mitochondrial division-related protein DRP1 (419). Mdivi-1’s suppression of mitophagy improves silibinin-mediated apoptosis in breast cancer (419) (**Table 2**).

**Table 2:** the effects of autophagy-mediated medications in different types of cancer and their mechanisms.

Category	Medication	Human/Animal/Cell Line	Effects on Autophagy/Mitophagy	Type of Cancer	Mechanism	Ref
**Autophagy Regulation as Side Effect**	Metformin	Shikawa human endometrial adenocarcinoma cell line A549, Calu-3 and HCC-15 lung cancer cell line	Autophagy Activation in Cancer Cell	• Endometrial Cancer • Lung Cancer	• Prevents Cell Viability and Proliferation • Promotes Apoptosis • Increasing the Levels of LC3-II • Decreasing the Levels of p62	(316, 403)
**Cancer Prevention**	Tamoxifen (Tam)	MCF7 cells	Autophagy Activation	Breast Cancer	• Overexpress Metallothionein 2A and Ferritin Heavy Chain mRNAs in MCF7-TamR Cells by Autophagy Inhibition	(328)
**Anti-Cancer Therapy**	ATG5 and Beclin-1 siRNA	A549 cells lung cancer cell line	Autophagy Inhibition	Lung Cancer	• Increasing Caspase Activity • Decreasing Cell Survival • Improves Cisplatin Sensitivity	(333)
	3-methyladenine (3-MA)	HCT116 cell line	Autophagy Inhibition	Colorectal Cancer	• Promoting Hypoxia • Inducing Apoptosis	(334)
	The Combination Therapy with the Reactivation of p53 and Induction of Tumor Cell Apoptosis (RITA) and 3-methyladenine (3-MA)	AMC-HN2–10	Autophagy Inhibition	Head and Neck Cancer	• Autophagy Suppression • Promotion of Apoptosis	(337)
	Enzalutamide (ENZ)	82, T24, and UMUC3 cell lines	Autophagy Stimulation	Bladder Cancer	• Increase in AMPK, ATG5, LC3B, and ULK1 Levels	(335)
	Atorvastatin (ATO) with an Autophagy Inhibitor, such as 3-MA or Baf A1	SiHa and Caski human cervical cancer cell lines	Autophagy Induction (as Side Effect)	Cervical Cancer	• Reduces Cancer Development • Stimulation of Apoptosis-Related Proteins such as Caspase-3, PARP, and Bim	(336)
**Baf A1**	Bafilomycin A1 (Baf A1) at Low Doses	RS4;11, NB4, HL-60, K562 and BV173 Leukemia cell lines	Autophagy Inhibition	B-cell Acute Lymphoblastic Leukemia (ALL Type)	• Apoptosis Augmentation • Targets Mitochondria	(339)
**Medications Affect mTOR Signaling**	Rapamycin	Mouse Model	Autophagy Inhibition	Lung Cancer	• Inhibiting PI3K-AKT-mTOR signaling	(348)
	Rapamycin at Low Doses	APCMin/+ mice	Autophagy Inhibition	Intestinal Neoplasia	• Increasing AKT-mTOR Signaling	(350)
	SAR405	GFP-LC3 HeLa cells	Autophagy Inhibition	Not Applicable	• Reducing PIK3C3’s Catalytic Activity	(355)
	SB02024	MCF-7 cells	Autophagy Inhibition	Breast Cancer	• Decrease Breast Cancer Xenograft Models • Work in Synergy with other Treatments *in vitro*	(356)
	ULK1 and VPS34 Genetic Inhibitors	BRAFi-sensitive and resistant AM38 and MAF794 cell lines	Autophagy Inhibition	Central Nervous System (CNS) Tumor Cells	• Not Applicable	(358)
	Quercetin (Natural Flavonol)	HG3 cell line	Autophagy Activation	Leukemic Cell Lines and B-Cells	• Inhibition of the PI3K/AKT Pathway • Multi-Kinase Inhibitor	(365)
	ABT-737 (ABT-263 and ABT199)	Cell Line	Inducing Autophagic-Like Cell Death	Glioblastoma	• Inhibit the Interaction Between Beclin-1 and Bcl2	(420)
	Flavonoid Isoliquiritigenin (ISL)	HCC MHCC97-H and SMMC7721 cells	Autophagy Boosting	Hepatocellular Carcinoma	• Altering the PI3K/AKT/mTOR Pathway • Increasing Apoptosis	(367)
**Main Medications in Autophagy Regulation**	Chloroquine (CQ)	RT4, 5637, and T24human bladder cell lines MiaPaCa2 (non-metastatic) and S2VP10 (metastatic) cell lines SW620 and HCT116 cells	Autophagy Inhibition	• Bladder Adenocarcinoma • Pancreatic Adenocarcinoma • Therapeutic Benefits of PS-PDT in Colorectal Cancer	• Altering Lysosomal pH • Stopping Autophagic Breakdown • Increasing Autophagosomal Accumulation • Increasing Apoptosis • Protonating and preventing Lysosome Diffusion • Decreasing Cancer Cell Proliferation in the Bladder and Pancreatic Adenocarcinoma	(369, 373, 379)
	Hydroxychloroquine (HCQ)	RT4, 5637, and T24human bladder cell lines MiaPaCa2 (non-metastatic) and S2VP10 (metastatic) cell lines Phase I clinical trial study	Autophagy Inhibition	• Bladder and Pancreatic Adenocarcinoma • Pancreatic Ductal Adenocarcinoma (PDAC) • Multiple Myeloma (MM)	• Altering Lysosomal pH • Stopping Autophagic Breakdown • Increasing Autophagosomal Accumulation • Increasing Apoptosis • Protonating and preventing Lysosome Diffusion • Decreasing Cancer Cell Proliferation in the Bladder and Pancreatic Adenocarcinoma	(369, 373, 390)
	Combination of HCQ with the Histone Deacetylase Inhibitor Vorinostat (VOR)	Phase I and II clinical trial study	Autophagy Inhibition	Metastatic Colorectal Cancer	• Not Applicable	(391)
	Everolimus (RAD-001)	HEC-1A cell lines	Autophagy Induction	Endometrial Cancer	• Increases Susceptibility to Paclitaxel-Induced Apoptosis • Downregulation of AKT/mTOR phosphorylation • Accumulation of LC3	(392)
**New Medications**	Lys05	HT-29 colon cells line 1205Lu and c8161 melanoma cell lines	Autophagy Inhibition	• Colorectal Adenocarcinoma • Melanoma	• affecting other Parts of the Autophagic Pathway	(395)
	DQ661	HT29 colorectal cell line A375P, 451Lu 1205Lu, C8161, WM1361A, and WM3918 melanoma cell lines	Autophagy Inhibition	• Colorectal Cancer • Melanoma	• Not Applicable	(394)
	Combination of DQ661 with Gemcitabine	KRPC, PDA.4662 and G43 pancreatic cancer cell lines	Autophagy Inhibition	Pancreatic Ductal Adenocarcinoma (PDAC)	• Not Applicable	(394)
	Combination of SAR405 with Everolimus	Renal tumor cell lines	Autophagy Inhibition	Suppression of Kidney Cancer Cell Growth	• Impairing Lysosomal Function • Interfering with the Late Endosome-Lysosome Interface	(410)
	Doxorubicin	Human keratinocyte HaCaT cells HCT8 human colorectal cancer cells	Mitophagy Inhibition	Colorectal Cancer	• DNA-Damaging Effect • Aberrant Mitochondrial Activity • Creation of Superoxide • Silencing BNIP3L	(411, 412)
	Mitochondrial Division Inhibitor 1 (mdivi-1)	MCF-7 and MDA-MB-231 cells	Mitophagy Inhibition	Breast Cancer	• Inhibition of Dynamin I and the Mitochondrial Division-Related Protein (DRP1) • Improves Silibinin-Mediated Apoptosis	(419)

Further research is necessary; however, other pharmacological autophagy activators could possibly be helpful for cancer chemoprevention. Most of these medications lack anticancer activity and selectivity (421). Given the many studies demonstrating that autophagy is triggered as a survival response to antineoplastic treatment, the bulk of the trials has combined HCQ with other antineoplastic regimens, such as chemotherapy, targeted therapy, and radiation therapy (386). As a result, new biomarkers and methodologies are needed (386). Autophagy inhibitory effects of CQ and HCQ in clinical studies have yet to be determined, even though these compounds have been shown beneficial *in vitro* (374). In addition, the pharmacokinetics of HCQ make it difficult to achieve appropriate micromolar dosage levels during short periods (422, 423). Targeting downstream ubiquitin-like conjugation machinery, including Atg7, maybe a potential therapeutic method (374). Autophagy plays a critical part in normal tissue homeostasis; therefore, potency and toxicity must be balanced (374).

## 10 Conclusion

In cancer biology, autophagy is crucial for the expansion and multiplication of cancer cells as well as the control of tumor formation (27, 28). The modulation of autophagy has the potential to impact the manifestation of tumor suppressor proteins or oncogenes. In addition, manipulating autophagy through specific anticancer drugs can potentially promote either the viability or demise of malignant cells (29, 424). Autophagy is a cellular process that is activated in response to stress or nutrient-deprivation conditions. This process is essential for maintaining cellular metabolism and can also be utilized to eliminate pathogens and apoptotic cells. Mutations in autophagy-related genes (ATGs) modulate autophagy and have been linked to various diseases affecting humans, such as cancer (19–21). The relationship between autophagy and malignancies has gained widespread recognition. However, the dualistic character of autophagy, which can act as either a promoter or inhibitor of tumor growth, has raised concerns regarding the regulation of tumorigenesis without triggering deleterious aspects of autophagy. The impact of autophagy on tumor cells at different stages (initiation, development, and progression) remains a complex and contentious phenomenon in the context of cancer therapies. Autophagy is believed to have a significant impact on the preservation of stemness in cancer stem cells, as well as the regulation of these cells’ homeostasis (40, 41).

It is of utmost importance to comprehend the mechanisms through which autophagy contributes to the development of cancer and the factors that dictate its pro- or antitumor impacts, in order to devise efficacious therapeutic approaches. Interventions targeting the modification of tumor cell fate through the regulation or induction of autophagy substrates necessitate meticulous monitoring of these mediators’ activities within the tumor microenvironment to enable informed decisions regarding their activation or deactivation. The investigation of the functioning of autophagy in cancer is a complicated and ongoing area of research. Additionally, there are many forms of autophagy that are solely applicable to particular cell organelles. There is a lot of autophagy research going on right now. These novel findings, however, bring up a lot of new issues. Therefore, additional research is required to understand the primary role of autophagy in various disorders, including cancer. However, there have been some recent successes.

Several research studies have been carried out, indicating a correlation between the removal of the BECN1 gene and the development of breast, ovarian, and prostate cancer (102, 103). Furthermore, it has been observed that animal models exhibiting deficient expression of this particular gene display heightened vulnerability to liver and lung tumors as well as lymphoma (57, 104). Furthermore, the upregulation of this particular gene has been associated with a decrease in cellular proliferation and the hindrance of tumorigenesis in cases of breast cancer (103). Mice that are hemizygous for BECN1 and those that lack Atg4C and BIF1 exhibit a heightened likelihood of tumor formation due to the absence of these autophagic factors (102, 103, 110). Additionally, mice with deficiencies in ATG5 and ATG7 are more prone to the development of liver tumors (105). The protein ATG5 has been identified as a potentially crucial modulator in the decision-making process of cells to undergo either autophagy or apoptosis (116, 119). The pathways of p53 and autophagy are closely interconnected and have a notable impact on stress, metabolism, and cancer reactions (120).

Autophagy can potentially supply nutrients to cancer cells and aid in their acclimatization to unfavorable surroundings. While autophagy has been found to inhibit tumorigenesis, it has also been observed to facilitate tumorigenesis and the progression of tumors. The twofold character of autophagy’s property necessitates additional exploration. The induction of hypoxia within the tumor microenvironment via various molecular and cellular mechanisms results in an upregulation of autophagic activity. This, in turn, leads to tumor proliferation via two distinct pathways.

Autophagy is closely associated with the survival and maintenance of cancer stem cells, which can result in the progression of tumors to secondary metastatic sites and resistance to therapy. The present data substantiates the concept that autophagy serves dual functions in the process of cancer metastasis, acting either as a facilitator or a suppressor, contingent upon the tumor cell category, the microenvironment of the tumor, and the stages of the metastatic cascade (12, 13). Although direct quantification of autophagic flux in primary human malignancies samples is currently unfeasible, various studies utilizing surrogate markers have indicated a correlation between increased autophagy and metastasis.

Autophagy, a cellular process, is essential for the proper functioning of immune responses that are both innate and adaptive (29). The autophagy process in tumor cells undergoing apoptosis is a crucial factor in the initiation of immunogenic cell death. This mechanism facilitates the efficient identification of tumors by the immune system, as reported in references (6, 155). The presentation of tumor antigens is a crucial component of anti-cancer responses. A breakdown in this mechanism may result in immune evasion by tumors, a phenomenon commonly associated with cancer progression. It has been observed that inhibiting autophagy may block this process (157, 425). Several studies have been conducted to establish the unique regulatory mechanisms of macroautophagy and CMA in peripheral T cells. Preliminary investigations suggest that the activation of macroautophagy in T lymphocytes entails distinct signaling pathways. Previous studies have indicated that the activation of the T cell receptor (TCR) results in an increase in macroautophagy in CD4+ and CD8+ T cells (158–161). The complete comprehension of the signaling mechanisms that trigger macroautophagy stimulation in active T cells is still underway (158). For T cell homeostasis to be maintained, macroautophagy is essential (158). The process of mitophagy-mediated mitochondrial recycling holds significant importance in T cells, as they undergo a substantial reduction in mitochondrial content during their maturation from immature peripheral naive T cells to single-positive thymocytes (158). Autophagy is a crucial mechanism that governs the production and dissemination of cytokines, including IL-1, IL-18, and TNF-α (187). Sufficient levels of reactive oxygen species (ROS) play a crucial role in facilitating autophagy, while the buildup of ROS within the tumor microenvironment impedes the ability of dendritic cells to defend against tumors (29, 214). The origin of this is primarily attributed to the respiratory chain located within the mitochondria (29). ROS has an impact on the entire tumor growth process (212). The process of mitophagy involves the elimination of mitochondria that have been impaired and are producing reactive oxygen species (ROS) within the cell (29).

Both inducing and inhibiting autophagy can have both beneficial and detrimental consequences. Autophagy inhibition is a defensive mechanism against chemotherapy, and it can also result in secondary diseases including various malignancies (154). Chemotherapeutic medicines such as etoposide, fenretinide, and dexamethasone produce autophagic cell death *in vitro* (308–311). Sodium selenite only affects malignant glioma cells via mitophagy resulting in cell death (313, 314). There exist pharmacological agents that do not primarily function to induce or inhibit autophagy, yet have demonstrated the ability to modulate autophagy. One such example is metformin, commonly used for its anti-diabetic properties. Conversely, the administration of ATO, a medication employed for the purpose of diminishing blood cholesterol concentrations, in conjunction with an autophagy inhibitor, has been observed to yield beneficial outcomes in the context of cancer therapy (287). There exists a potential for certain pharmacological agents, including tamoxifen, to impede the proliferation of cancer cells via the augmentation of autophagy (328). However, the precise signaling pathways that elucidate the connection between autophagy and endocrine resistance remain incompletely characterized (328). This topic holds significant potential for future academic investigation. Baf A1 exhibits a reduction of autophagy and enhancement of apoptosis in the treatment of cancer (287). The dysregulation of PI3K-AKT-mTOR signaling has been observed in various types of human cancers (347). The administration of rapamycin, an inhibitor of the mTOR, in a mouse model was found to be associated with a significant reduction of 90% in the incidence of lung cancers induced by carcinogens (348). CQ and HCQ directly inhibited autophagy (370, 371). The combination therapy of HQC and QC for cancer treatment is commonly employed, with a focus on the regulation of autophagy. Novel inhibitors with improved selectivity and efficacy are currently under development, specifically targeted toward the next generation’s lysosomes. These inhibitors have demonstrated enhanced anti-cancer properties.

More research is required to understand the molecular mechanisms that govern autophagy in tumor initiation and progression. In view of the complexity of signaling pathways involved, such an extensive characterization of the role of autophagy in cancer should be instrumental in providing insights about therapeutic methods. A more thorough understanding of these mechanisms will help develop targeted therapies that can also overcome the resistance and improve clinical outcomes.

## 11 Future prospect

Potential avenues for future research in the realm of autophagy and cancer may encompass a variety of domains such as:

1. Additional research is required to comprehensively comprehend the molecular mechanisms underlying autophagy, which plays a role in the initiation, advancement, and progression of cancer pathogenesis. This information has the potential to offer valuable insights into new therapeutic targets for the treatment of cancer. Moreover, clinical trials are being conducted to examine the effectiveness and safety of autophagy-targeted therapies in diverse cancer patients. The aim is to investigate the potential of such therapies in treating cancer. The aforementioned studies have the potential to furnish significant insights regarding the clinical efficacy of therapies that target autophagy in the context of cancer treatment.
2. For investigating the function of autophagy within the context of the tumors’ microenvironment. The examination of autophagy’s function within the tumor microenvironment, including its impact on tumor suppression, promotion, and interaction with the immune system, may yield valuable information regarding potential therapeutic targets for various forms of cancer.

Future research endeavors should investigate the feasibility of amalgamating autophagy-focused therapeutic interventions with alternative treatment modalities, such as chemotherapy and immunotherapy, to augment their effectiveness and surmount pharmacological resistance.

To summarize, potential avenues for future research in the realm of autophagy and cancer could lead to significant revelations regarding innovative therapeutic targets, treatment methodologies, and the impact of autophagy on cancer, ultimately leading to enhanced outcomes for cancer patients.

## Glossary

ALL: Acute lymphoblastic leukemia
AMBRA1: Activating molecule in Beclin-1-regulated autophagy
AMPK: AMP-activated protein kinase
APC: Antigen-presenting Cells
AS2O3: Arsenic trioxide
ATG: Autophagy-related gene
ATO: Atorvastatin
BAF A1: Bafilomycin A1
BCA-m: Bacillus caldovelox arginase mutant
BNIP3: Bcl-2/adenovirus E1 19kDa interacting protein 3
BNIP3L: BNIP3-like protein
CD: Crohn’s disease
CMA: Chaperone-mediated autophagy
CQ: Chloroquine
DC: Dendritic Cell
ECM: Extracellular matrix
EGFR: Epidermal growth factor receptor
EMT: Epithelial-mesenchymal transition
ENZ: Enzalutamide
FAK1: Focal adhesion kinase 1
GEMMs: Genetically engineered mouse models
GBM: Glioblastoma
HCQ: Hydroxychloroquine
HEK293T: Human embryonic kidney cell lines
HMGB1: High mobility group box 1
HNK: Honokiol
HIF-1: Hypoxia-inducible factor 1
H4K16: H4 lysine 16
H4K16ac: H4K16 acetylation
IBC: Isobavachalcone
IL6: Interleukin 6
ISL: Isoliquiritigenin
LAMP-2A: Lysosomal associate membrane protein 2A
LAP: LC3-associated phagocytosis
LC3B: Light chain 3B
MAPK: Mitogen-activated protein kinase
mTOR: Mammalian target of rapamycin
MDM2: Mouse double-minute 2 homolog
MMP2: Matrix metalloproteinase-2
mPFS: Median progression-free survival
MHC: Major histocompatibility complex
NBR1: Neighbor of BRCA1 gene 1
NFAT: Nuclear factor of activated T cells
NF-B: Nuclear Factor-B
NK: Natural Killer
NSCLC: Non-small cell lung cancer
OS: Overall Survival
OXPHOS: Oxidative phosphorylation
PARP: Poly ADP-ribose polymerase
PC: Plasma cells
PDAC: Pancreatic ductal adenocarcinoma
PE: Phosphatidylethanolamine
PI3KC3: Class III Phosphatidylinositol 3 Kinase Complex
PKC: Protein kinase C
REDD1: Regulated in development and DNA damage response 1
RITA: Reactivation of p53 and induction of tumor cell apoptosis
ROS: Reactive oxygen species
SBI: SBI-0206965
TAM: Tamoxifen
TCR: T cell receptor
TFEB: transcription factor EB
TMZ: Temozolomide
UBA: ubiquitin-associated
ULK1: Unc-51-like kinase 1
VDAC1: Voltage-dependent anion channel 1
VOR: Vorinostat
WNT5A: Wnt family member 5a
3-MA: 3-methyladenine
